# A critical role of PvFtsH2 in the degradation of photodamaged D1 protein in common bean

**DOI:** 10.1038/s41438-021-00554-7

**Published:** 2021-06-01

**Authors:** Kun Xu, Jinlong Zhu, Hong Zhai, Hongyan Wu, Yi Gao, Yuzhuo Li, Xiaobin Zhu, Zhengjun Xia

**Affiliations:** 1 Key Laboratory of Soybean Molecular Design Breeding, Northeast Institute of Geography and Agroecology, The Innovative Academy of Seed Design, Chinese Academy of Sciences, Haping Road 138, Nangang District, 150081 Harbin City, Heilongjiang Province China; 2 University of Chinese Academy of Sciences, Beijing, China

**Keywords:** Photosynthesis, Agricultural genetics

## Abstract

Light is required for initiating chloroplast biogenesis and photosynthesis; however, the photosystem II reaction center (PSII RC) can be photodamaged. In this study, we characterized *pvsl1*, a seedling-lethal mutant of *Phaseolus vulgaris*. This mutant showed lethality when exposed to sunlight irradiation and a yellow-green leaf phenotype when grown in a growth chamber under low-light conditions. We developed 124 insertion/deletion (INDEL) markers based on resequencing data of Dalong1 and PI60234, two local Chinese common bean cultivars, for genetic mapping. We identified *Phvul.002G190900*, which encodes the PvFtsH2 protein, as the candidate gene for this *pvsl1* mutation through fine-mapping and functional analysis. A single-base deletion occurred in the coding region of *Phvul.002G190900* in the *pvsl1* mutant, resulting in a frameshift mutation and a truncated protein lacking the Zn^2+^ metalloprotease domain. Suppressed expression of *Phvul.002G190900* at the transcriptional level was detected, while no change in the subcellular localization signal was observed. The seedlings of *pvsl1* exhibited hypersensitivity to photoinhibition stress. In the *pvsl1* mutant, abnormal accumulation of the D1 protein indicated a failure to rapidly degrade damaged D1 protein in the PSII RC. The results of this study demonstrated that PvFtsH2 is critically required for survival and maintaining photosynthetic activity by degrading photodamaged PSII RC D1 protein in common bean.

## Introduction

Plants suffer from various stresses, such as high-intensity light. Enhanced stress tolerance is vital for plants to survive and adapt to environmental changes. Light drives chloroplast biogenesis^1,2^. Chloroplasts are the primary location for photosynthesis, and the products of photosynthesis provide humans with food and oxygen^3^. In light, ^1^O_2_, a known byproduct of photosynthesis, constantly damages PSII RC protein D1. Heat, nitric oxide, macronutrient stress, and pathogens can also influence D1 protein levels^4,5^. Plants have evolved the PSII repair cycle, in which filamentation temperature sensitive H (FtsH) metalloprotease plays a central role in the degradation of photodamaged D1 protein over time^6,7^. D1 degradation, which proceeds from the N-terminus by FtsH, is fine-tuned by PSII core phosphorylation to avoid undesirable cleavage^8^. FtsH consists of an N-terminal transmembrane domain, an AAA domain (ATPase associated with various cellular activities), and a Zn^2+^ metalloprotease domain^9^. The mutation of *FtsH2* leads to an attenuated ability to deal with photoinhibition, resulting in leaf variegation in Arabidopsis^10,11^. There are several allelic mutations at the *FtsH2* locus, including *var2-1* (a nonsense mutation, Q597*), *var2-2* (a missense mutant, R191K), *var2-3* (a missense mutation, G267D), *var2-4* (a T-DNA insertion mutation, SAIL_253_A03), and *var2-5* (a missense mutation, P320L)^12,13^. The *var2-1* allele, the most severe allele^14,15^, showed a variegated phenotype under illumination of 60 μmol·m^−2^·s^−1^. The *var2-5* allele, the weakest allele, displayed leaf variegation and significantly reduced leaf chlorophyll content under illumination of ~100 μmol·m^−2^·s^−1^ ^14^. The increase in light intensity was associated with leaf symptoms in *var2* mutants. For example, the leaves of *var2-4* mutants showed green and yellow sectors when grown under illumination of 80 μmol·m^−2^·s^−1^ but showed green and white sectors under ~100 μmol·m^−2^·s^−1^ illumination^14,16^. *FtsH2* is particularly important for seedling survival in Arabidopsis both in a controlled environment and under field conditions^17^. However, the functions of the *FtsH* gene family have not been well elucidated in crop species.

Artificial mutagenesis generally creates rich genetic variations for studying gene function^18,19^. We characterized a recessive lethal mutation, referred to as *pvsl1*, from a mutant library of Dalong1 generated via gamma radiation. We fine-mapped the *PvSL1* locus to a 53.7 kb region on chromosome 2. We concluded that *Phvul.002G190900*, encoding the PvFtsH2 protease, is the causal gene for the *PvSL1* locus. In the field, *pvsl1* seedlings died ~2 weeks after germination. However, when grown in a growth chamber under low light, *pvsl1* seedlings could survive, flower, and set seeds even though a yellow-green leaf phenotype was observed. *pvsl1* plants displayed an impaired capacity to defend against photoinhibition. The results of this study demonstrate the importance of *PvFtsH2* in maintaining photosynthetic activity by degrading the D1 protein in common bean.

## Results

### The *pvsl1* mutant displays a lethal phenotype under sunlight irradiation

**Fig. 1:**
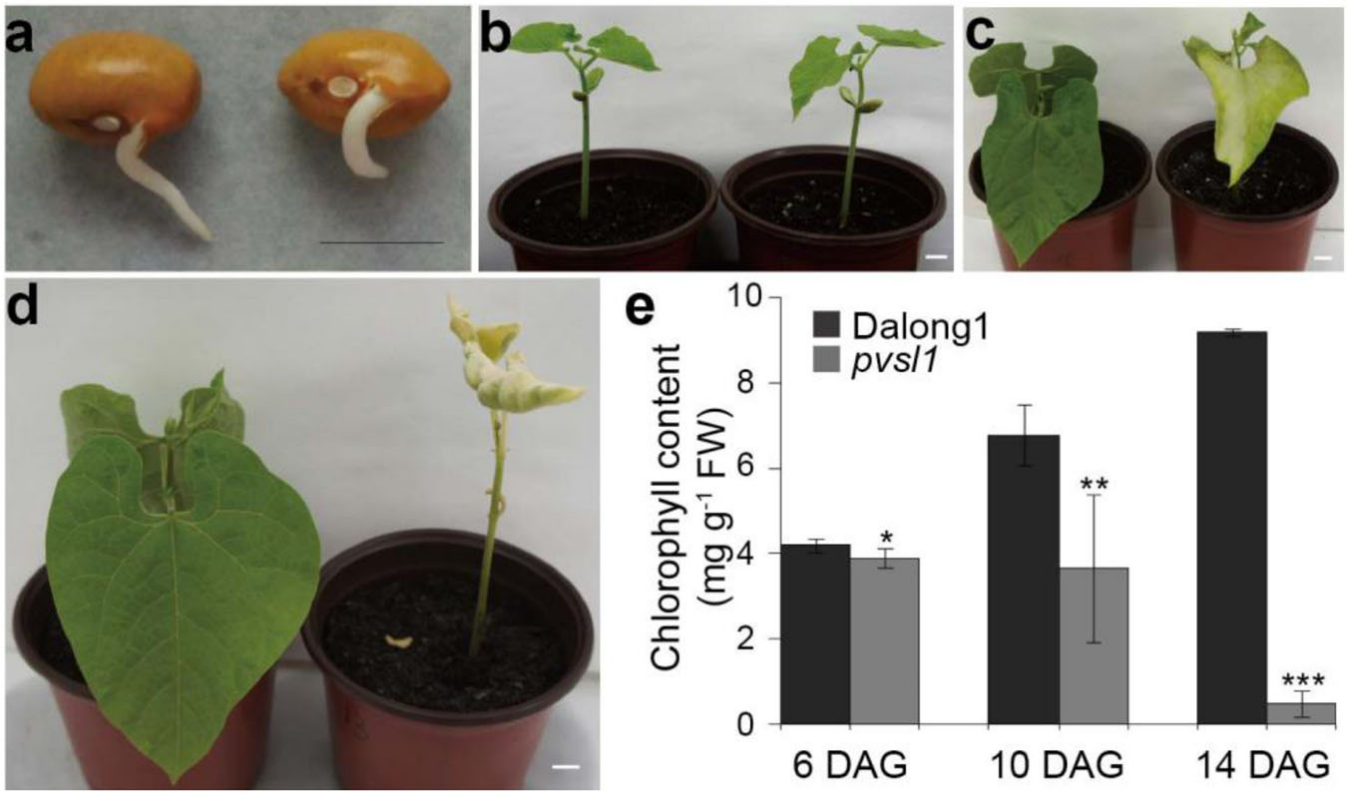
Phenotypes of the pvsl1 mutant. **a**–**d** Phenotypes of Dalong1 (left) and *pvsl1* (right) seeds after germination. **a** 2 DAG (days after germination); **b** 6 DAG; **c** 10 DAG; **d** 14 DAG. Bars = 1 cm. **e** Chlorophyll contents of Dalong1 and *pvsl1* plants measured at 6, 10, and 15 DAG. At least 10 seedlings per treatment were measured. Error bars indicate SD

Common bean (*Phaseolus vulgaris L*.) is one of the most important legume crops in the world. This species has a relatively small genome^20^ of ~587 Mb and a short life cycle. To date, gene function studies in common bean have rarely been reported. Aiming to create rich genetic variations for studying gene function, we developed a mutant library by exposing seeds of the common bean cultivar Dalong1 of the golden hook ecotype to ^60^Co radiation at a dosage of 200 Gy. Phenotypic observation was performed in the M3 generation. Among 30 plants of line M138 derived from a single M2 plant, 22 plants showed a normal wild-type phenotype, while eight plants showed a seedling-lethal phenotype, referred to as *pvsl1*. In the field, the *pvsl1* mutant seedlings died ~2 weeks after germination (Fig. S1). When grown in pots under sunlight irradiation, the germination and emergence of cotyledons and unifoliate leaves showed no noticeable difference between the *pvsl1* mutant and Dalong1 (Fig. 1a, b). However, the tender unifoliate leaves of *pvsl1* started to show a light to more apparent yellow-green color compared with Dalong1 (Fig. 1c). Approximately 10 days after germination (DAG), the leaves of the *pvsl1* mutant withered, and the whole plant eventually died 14 DAG (Fig. 1d). Considering that chlorophyll content is an important indicator of leaf color, we measured the chlorophyll content in the leaves of *pvsl1* and Dalong1. Consistent with leaf color appearance, a significant reduction in chlorophyll content in the *pvsl1* mutant was observed starting from 6 days after germination when compared with that in Dalong1 (Fig. 1e).

### Map-based cloning of *PvSL1*

To identify the gene controlling the seedling-lethal phenotype in the *pvsl1* mutant, we constructed segregation populations by crossing PI60234 as the female parent with M138 showing a nonlethal phenotype and possibly carrying heterozygous *pvsl1* as the male parent. All five F1 plants showed the nonlethal phenotype. We observed that the *pvsl1* seedling-lethal mutation was segregated in one F2 population and that the segregation ratio agreed with the normal 3:1 Mendelian ratio (Table S1), indicating that the lethal phenotype of the *pvsl1* mutant was controlled by a single recessive nuclear gene.

The genomes of two parent cultivars, Dalong1 and PI60234, were resequenced to develop insertion/deletion (INDEL) markers for genetic mapping. After being trimmed by NGSQCToolkit (version of v2.3.3)^21^, INDELs and SNPs between Dalong1 and PI60234 were called using SpeedSeq^22^. These variations were further checked manually using an integrative genomics viewer (IGV version 2.5.0)^23^. We developed molecular markers targeting 165 candidate INDEL sites whose polymorphisms could be visualized by agarose gel electrophoresis. Among the 165 candidate INDEL sites, 124 markers were confirmed to be polymorphic between Dalong1 and PI60234 by PCR tests and hence were used for genetic mapping. The 124 INDEL markers were randomly distributed among 11 chromosomes of the common bean genome (Fig. S2). The number of INDEL markers ranged from 5 to 22 per chromosome. INDEL marker information, including primer sequences and physical position, is provided in Supporting Information, Table S2.

**Fig. 2:**
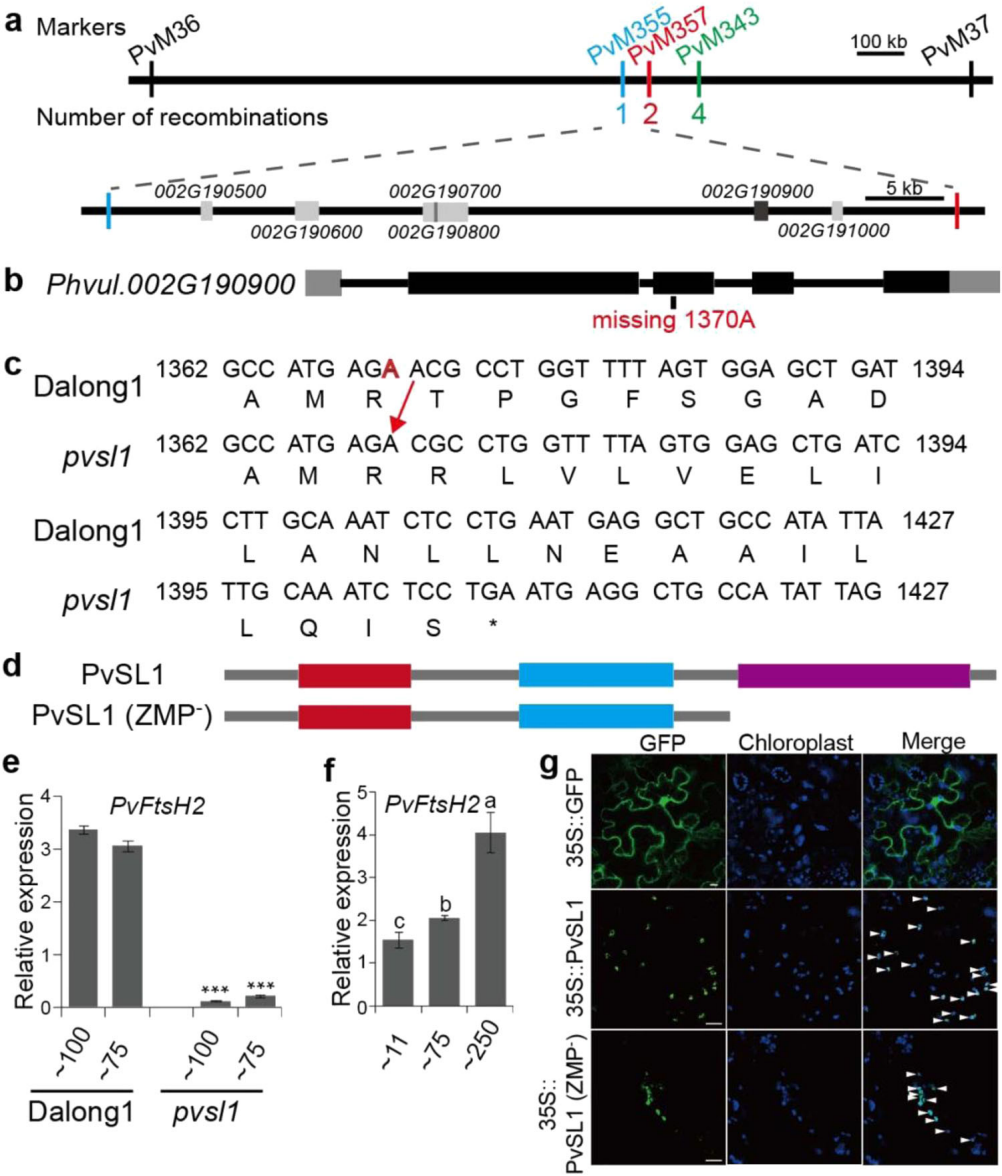
Map-based cloning of the *PvSL1* locus. Using 124 INDEL markers, the *PvSL1* locus was mapped to a 1675.3 kb region between PvM36 and PvM37 on chromosome 2. **a** Physical locations of fine-mapping markers and the number of recombinants defining the *PvSL1* region. The *PvSL1* locus was fine-mapped to the region between PvM359 and PvM357, in which six genes (*Phvul.002G190500* to *Phvul.002G191000*) were annotated. **b**, **c** In the *pvsl1* mutant, a single base was deleted at 1370 bp (A1370) in the third exon of *Phvul.002G190900*, leading to a frameshift mutation. In the gene model, exons, introns and UTRs are represented by black boxes, solid lines, and gray boxes, respectively. **d** The mutated Phvul.002G190900 protein retained the FtsH extracellular domain (red box) and ATPase domain (blue box) but lacked the Zn^2+^ metalloprotease domain (purple box) (Protease). **e** Expression levels of *PvSL1* in Dalong1 and mutants under 100 and 75 μmol·m^−2^·s^−1^ light conditions. **f** Expression levels of *PvSL1* under 11, 75, and 250 μmol·m^−2^·s^−1^ light conditions. **e**, **f** Unifoliate of 10-day-old seedlings were used to extract total RNA. *Phvul.007G041700.1*, the homologue of *TIP41* in common bean, was used as the reference gene. Error bars indicated SD. **g** Subcellular localization of the PvSL1 and PvSL1 (ZMP^−^) proteins. Bars were 10 μm

Using the 124 INDEL markers, we initially mapped the *PvSL1* locus to a 1675.3 kb genomic region between PvM36 and PvM37 on chromosome 2 using individuals from an F2 population (Fig. S3 and Table S3). Subsequently, we further developed three new INDEL markers (PvM355, PvM357, and PvM343) targeting the polymorphisms between PvM36 and PvM37 (Table S4). Nine recombinants whose recombination occurred between PvM36 and PvM37 were identified from 200 individuals of F2:3 populations derived from heterozygous F2 plants, enabling us to further delimit the *PvSL1* locus (Fig. 2a). As a result, the *PvSL1* locus was delimited to a 53.7 kb region between PvM355 and PvM357, in which six genes (*Phvul.002G190500*, *Phvul.002G190600*, *Phvul.002G190700*, *Phvul.002G190800*, *Phvul.002G190900*, and *Phvul.002G191000*) were annotated according to the common bean reference genome (Fig. 2a). Based on the resequencing data, we compared the genetic variations between Dalong1 and the *pvsl1* mutant that occurred in the delimited region using IGV^23^. Interestingly, only a single-base deletion occurred in the coding region of *Phvul.002G190900*, leading to a truncated protein, while the remaining five genes showed no genetic variation at all (Fig. S4). We further checked the genomic region from Chr02:32373000 to Chr02:37870500 (*phvul.002G168200* to *phvul.002G211600*), which included 200 annotated genes on either side of *Phvul.002G190900*, and no other genetic variations occurred in the coding regions between Dalong1 and the *pvsl1* mutant on IGV^23^.

First, we validated whether *Phvul.002G190900* was the causal gene responsible for the seedling-lethal phenotype at the genetic level. All 39 plants, among 182 M5 plants derived from a single M4 plant carrying heterozygous *PvSL1* locus, showing *pvsl1* lethal-seedling phenotypes were identified to carry homozygous single-base deletion by sequencing of PCR products fitting a classical 3:1 Mendelian segregation ratio. For further validation, progeny populations derived from plants carrying homozygous wild-type, heterozygous, and homozygous mutated genotypes of *Phvul.002G190900* were generated. A wild-type phenotype was observed for all 150 plants from the populations derived from five plants carrying the homozygous wild-type genotype, while the *pvsl1* seedling-lethal phenotype was observed for 26 plants from the populations derived from five plants with homozygous mutated genotypes (the *pvsl1* seedlings could survive, flower, and set seeds when grown in a growth chamber, see below). Among the 150 individuals from the populations derived from five heterozygous individuals, we identified 116 plants showing the wild-type phenotype and 34 plants displaying the lethal phenotype, in agreement with the normal 3:1 Mendelian ratio for a single recessive gene.

A single-base deletion (A1370) occurred in the 3rd exon of *Phvul.002G190900* in *pvsl1* mutants, resulting in a frameshift mutation (Fig. 2b, c). The truncated protein had 447 amino acids (Fig. 2c, d). *Phvul.002G190900* was annotated as a member of the FtsH metalloprotease family in the reference genome. The Phvul.002G190900 protein in the *pvsl1* mutant, referred to as PvSL1 (ZMP^−^), lacked the Zn^2+^ metalloprotease domain (http://smart.embl.de/) since 250 amino acids at the C-terminus were truncated (Fig. 2d). The expression of *Phvul.002G190900* was significantly suppressed in the *pvsl1* mutant, which may be ascribed to the nonsense mutations often destabilizing RNA (Fig. 2e). The transcriptional abundances of *Phvul.002G190900* appeared to be associated with light intensity. Enhancing the strength of illumination obviously increased the expression level (Fig. 2f). The subcellular localization of PvSL1 (ZMP^−^) was similar to that of PvSL1 because PvSL1 (ZMP^−^) retained the functional chloroplast localization signal (Fig. 2g). However, the fluorescence signal of GFP-fused PvSL1 (ZMP^−^) was weaker than that of GFP-fused PvSL1, suggesting that the PvSL1 (ZMP^−^) protein might be unstable (Fig. 2g).

### Phylogenetic analysis of PvFtsH2 family genes from common bean and Arabidopsis

**Fig. 3:**
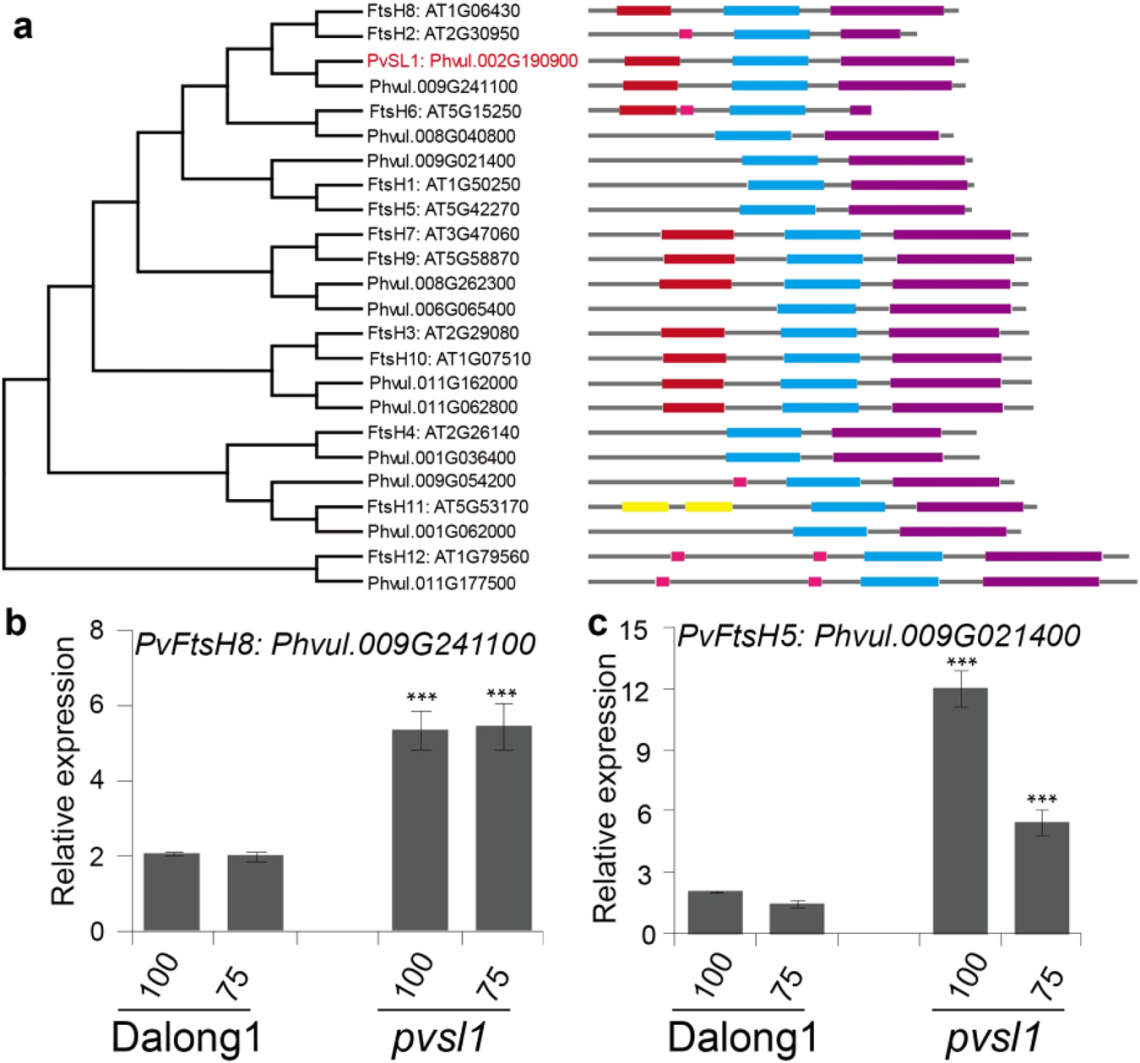
The PvSL1 protein is a member of the FtsH protease family in common bean. **a** Neighbor-joining phylogenetic tree of FtsH family proteins in Arabidopsis and common bean. Protein sequences were retrieved from phytozome (https://phytozome-next.jgi.doe.gov/info/Pvulgaris_v2_1). FtsH family members have an ATPase domain (blue box) and a Zn^2+^ metalloprotease domain (purple box) (http://smart.embl.de/). **b**, **c** The expression levels of *Phvul.009G241100* and *Phvul.009G021400* in Dalong1 and *pvsl1* under 100 and 75 μmol·m^−2^·s^−1^ light conditions. Unifoliate of 10-day-old seedlings were used to extract total RNA. *Phvul.007G041700.1*, homologous to *TIP41* in common bean, was used as a reference gene. Error bars indicate SD

The protein encoded by the *PvSL1* locus, *Phvul.002G190900*, is homologous to FtsH2 and belongs to the FtsH protease family (Fig. 3a). Both Arabidopsis and common bean genomes have 12 FtsH protease family members. PvSL1 has an ATP-binding domain and a Zn-binding domain, both of which are conserved among FtsH protease members. Phylogenetic analysis demonstrated that both PvSL1 and Phvul.009G241100 were close to Arabidopsis FtsH2 and FtsH8. PvSL1 displayed the highest amino acid identity to FtsH2 when comparing the protein sequence similarity, so *PvSL1* was referred to as *PvFtsH2* (Table S5). The expression of *Phvul.009G241100*, corresponding to *PvFtsH8*, was significantly increased in the *pvsl1* mutant (Fig. 3b). It was reported that both *FtsH2* and *FtsH5* jointly regulated the repair of damaged PSII; deletion of either *FtsH2* or *FtsH5* resulted in increased accumulation of the other at the protein level^24^. Phylogenetic analysis demonstrated that Phvul.009G021400 was homologous to FtsH5. We detected that the expression of *PvFtsH5* was significantly increased in the *pvsl1* mutant, especially under high-light intensity (Fig. 3c), suggesting that *PvFtsH2* and *PvFtsH5* might also jointly regulate the repair of damaged PSII in common bean. *PvFtsH8* expression levels were increased in the *pvsl1* mutant; however, *PvFtsH8* expression did not fluctuate with light intensity in either the *pvsl1* mutant or Dalong1 (Fig. 3b, c).

### The *pvsl1* mutant shows an impaired capacity to defend against photoinhibition

**Fig. 4:**
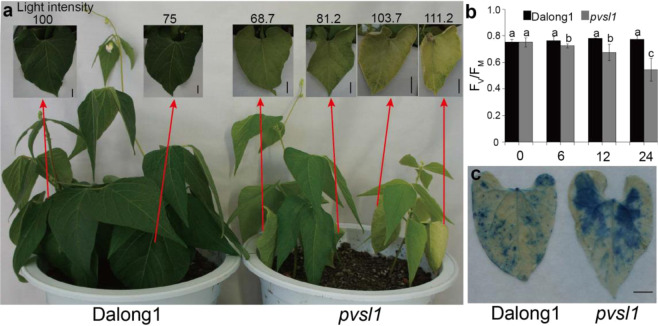
*PvSL1* plays a role in repairing photodamage. **a** Phenotypes of Dalong1 and *pvsl1* plants grown in an incubator under 68–112 μmol·m^−2^·s^−1^ light conditions. Higher light led to more chlorosis in *pvsl1* leaves. **b** Time-course (hours) analysis of the rate of PSII damage. *Pvsl1* and Dalong1 seedlings were initially grown under LD (25 μmol·m^−2^·s^−1^ at 22 °C) for 10 days before being subjected to photoinhibition conditions (100 μmol·m^−2^·s^−1^ at 18 °C, continuous light). PSII activity (Fv/Fm) was determined at the indicated time points. At least 10 seedlings per genotype were used for each measurement. Error bars indicate SD. **c** Trypan blue staining showed that more leaf cells were dead in *pvsl1*. Bars represent 1 cm

The *pvsl1* mutant could survive, flower, and set seeds under low light (Fig. S5). When grown in an incubator at light intensities of 100, 75, and 25 μmol·m^−2^·s^−1^, *pvsl1* leaves displayed yellow-green and marginal wilting phenotypes or slightly chlorotic phenotypes (Figs. 4a and S6a). Next, we determined whether the *pvsl1* mutant was susceptible to photoinhibition. PSII activity (*F*_*v*_*/F*_*m*_; *F*_*v*_, variable fluorescence; *F*_*m*_, maximum fluorescence) was used to reflect the levels of photoinhibition. It was determined that lowering the temperature at high-light intensity was an effective way to inhibit PSII activity in seedlings initially grown under low-light and normal temperature conditions^25^. Before being transferred to photoinhibition conditions (100 μmol·m^−2^·s^−1^ at 18 ± 2 °C), the *pvsl1* mutant and wild-type seedlings were initially grown under low-light intensity conditions (25 μmol·m^−2^·s^−1^ at 25 ± 2 °C) for 10 days. While no difference in *F*_*v*_*/F*_*m*_ was detected between Dalong1 and *pvsl1* plants grown under low-light intensity conditions, the *pvsl1* mutant seedlings showed an obvious decline in PSII activity within 12 h of being transferred to photoinhibition conditions (Fig. 4b). By visualization with trypan blue staining, a significant cell death response was observed in the *pvsl1* mutant compared to Dalong1 under photoinhibition conditions (Fig. 4c), suggesting that the *pvsl1* mutant displayed an impaired capacity to defend against photoinhibition. Taken together, our results demonstrate that *PvSL1* participates in defending against photodamage during leaf development and is required for the growth and survival of common bean when exposed to sunlight.

### The *pvsl1* mutant displays abnormal accumulation of the D1 protein under high-light intensity

**Fig. 5:**
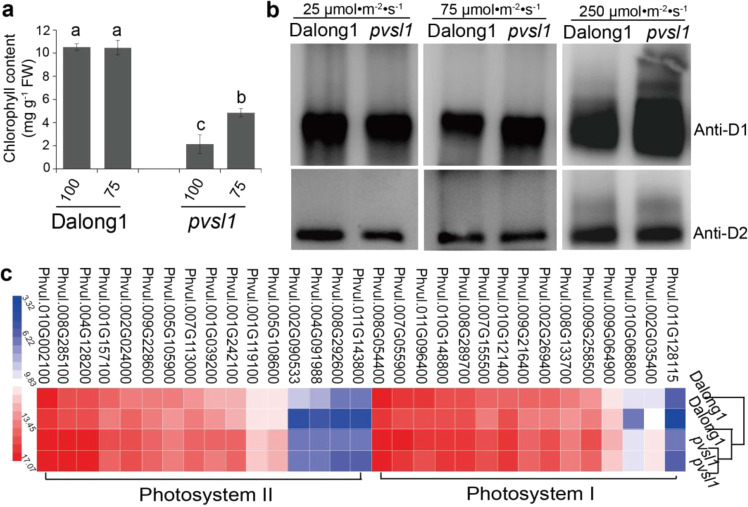
The function of FtsH protease was impaired in the *pvsl1* mutant. **a** Measurement of the chlorophyll content of Dalong1 and *pvsl1* mutant growing under 100 and 75 μmol·m^−2^·s^−1^ conditions. **b** Under low-light intensity conditions, 25 μmol·m^−2^·s^−1^, D1 protein accumulation was similar in the *pvsl1* mutant and Dalong1. However, under high-light intensity conditions, 75 μmol·m^−2^·s^−1^ and 250 μmol·m^−2^·s^−1^, D1 protein abnormally accumulated in the *pvsl1* mutant. D2 protein was used as a control. **c** Photosynthesis-related genes (photosystem I and photosystem II) were upregulated in the *pvsl1* mutant

We measured the chlorophyll contents of unifoliate of *pvsl1* and Dalong1 grown under different light intensities to evaluate the effect of light intensity on photosynthesis. The chlorophyll content was slightly decreased under low-light intensity (25 μmol·m^−2^·s^−1^) but dramatically reduced under higher light intensity (75 μmol·m^−2^·s^−1^ and 100 μmol·m^−2^·s^−1^) in the *pvsl1* mutant (Figs. 5a and S6b), in agreement with the leaf color appearance (Figs. 4a and S6a). FtsH complexes play roles in degrading the photodamaged PSII RC D1 protein but not the D2 protein^24,26–29^. We measured the D1 protein levels under low and high-light intensity conditions in both Dalong1 and *pvsl1* to evaluate the role of the PvFtsH2 protease in D1 protein accumulation. The results showed that the D1 protein levels were similar under low-light intensity conditions (Fig. 5b). However, under high-light intensity conditions, the D1 protein accumulation in *pvsl1* was greater than that in Dalong1 (Fig. 5b). The higher the light intensity was, the more D1 protein accumulated (Fig. 5b). These data suggested that photodamaged D1 protein could not be degraded rapidly to allow the turnover (coordinated degradation and synthesis) of D1 protein to repair the photodamaged PSII under light in the *pvsl1* mutant.

Transcriptional profiling under sunlight irradiation showed that some photosynthesis-related genes operating in PSI and PSII were upregulated in the *pvsl1* mutant (Fig. 5c and Table S6), suggesting that physiological changes occurred in response to strong photoinhibition. In Arabidopsis, FtsH2 functions by balancing cytosolic and chloroplastic translation^14^. We detected that a large proportion of genes associated with cytosolic and plastic ribosomal proteins were reduced in the *pvsl1* mutant by transcriptome analysis, suggesting that cellular proteome homeostasis is impaired (Tables S6 and S7). The homeostatic imbalance in the *pvsl1* mutant likely resulted in elevated expression of ubiquitination-related genes to degrade obsolete or even harmful proteins (Table S6). We conclude that PvFtsH2 is critically required for survival, maintaining photosynthetic activity and cellular proteome homeostasis in common bean.

## Discussion

### The applicability of INDEL markers used in gene cloning in common bean

We developed INDEL markers for genetic mapping based on whole-genome resequencing of parental cultivars with the aid of NGS software such as SpeedSeq^22^ and IGV^23^. Using 124 INDEL markers developed between Dalong1 and P61234, we successfully mapped the responsible gene underlying *pvsl1* to a 1675.3 kb genomic region on chromosome 2 in this study. Additionally, by using these INDEL markers, we successfully mapped other genes controlling sterility and dwarfism in populations having genetic background of Dalong1 mutants X P61234 in our laboratory. If the causal gene has been initially mapped through QTL mapping, MutMap, or other approaches, more saturated markers need to be developed for fine-mapping in the postgenome era. A similar strategy has been well documented for cloning genes responsible for various mutations in many crops, e.g., soybean^18^ and rice^19^. Furthermore, genetic variations in or adjacent to the delimited regions are also crucial for verifying the candidacy of the causal gene. In this study, segregation patterns of populations derived from heterozygous *PvSL1*/*pvsl1* as well as an F2 population derived from *PvSL1*/*pvsl1* X PI60234 crossing supported that *Phvul.002G190900* is the causal gene for *PvSL1*. Additionally, the candidacy of *Phvul.002G190900* was validated because no other genetic variations between the *pvsl1* mutant and Dalong1 occurred in the CDSs of genes in the 5.5 Mb region (Chr02:32373000 to Chr02:37870500).

### The importance of PvFtsH2 for survival and maintaining photosynthetic activity in common bean

**Fig. 6:**
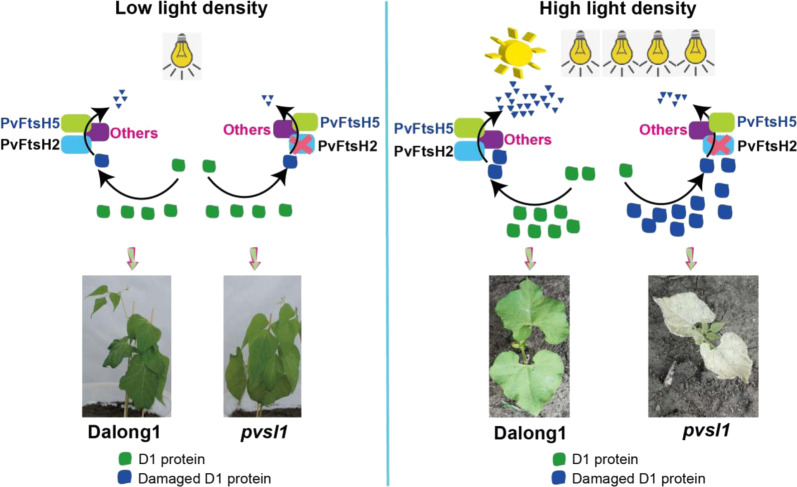
A model of the PvFtsH2-mediated response to photoinhibition through the degradation of damaged D1 protein. In common bean, the degradation of damaged D1 protein is mainly dependent on PvFtsH2. Grown under low-light intensity conditions, other PvFtsH members, e.g., PvFtsH5, may be capable of degrading damaged D1 protein in the *pvsl1* mutant. As a result, the *pvsl1* mutant could survive, flower, and set seeds (left). However, when grown under high-light intensity or sunlight conditions, a large amount of photodamaged D1 protein could not be degraded without functional PvFtsH2 in *pvsl1* mutants. Massive accumulation of photodamaged D1 protein may lead to a malfunctional photodamage-repair cycle of PSII and seedling lethality in *pvsl1* mutants (right)

Arabidopsis chloroplast FtsH tends to be a heterohexamer that includes ‘type B’ (FtsH2 and FtsH8) and ‘type A’ (FtsH1 and FtsH5) subunits^30^. In Arabidopsis, mutation of *FtsH2* results in a yellow variegated leaf phenotype^31,32^. Both FtsH2 and FtsH5 jointly regulate the repair of damaged PSII; deletion of either *FtsH2* or *FtsH5* led to the accumulation of more of the other protein^24^. In this study, we concluded that *Phvul.002G190900*, encoding the PvFtsH2 protein in common bean, is the causal gene for the *PvSL1* locus. The *pvsl1* mutant showed a deleterious phenotype in simple leaves as early as 10 DAG and died at ~14 DAG in the field or under high-light intensity conditions (Figs. 1d and S1). However, under low or medium light intensity conditions, normal growth or yellow-green leaf phenotypes were observed in the *pvsl1* mutant. *Phvul.009G021400* and *Phvul.009G241100*, corresponding to *PvFtsH5* and *PvFtsH8*, were significantly increased in the *pvsl1* mutant. We assume that PvFtsH5 and PvFtsH8 might also have potential effects on the D1 protein level. However, further studies are needed to reveal the functions of each PvFtsH as well as their coordinate relationship. Here, we present a model in which PvFtsH2 plays a central role in the turnover of the photosystem II D1 protein in common bean (Fig. 6). Although PvFtsH2 is nonfunctional, other PvFtsH family members, e.g., PvFtsH5 and PvFtsH8, may potentially be functional in degrading a limited amount of damaged D1 protein. However, seedling lethality under high-light intensity or sunlight conditions may reflect that a large amount of photodamaged D1 protein could not be degraded without a functional PvFtsH2 in *pvsl1* mutants. Taken together, we assume that *PvFtsH2* and other *PvFtsHs* might jointly regulate the repair of damaged PSII in common bean. Furthermore, the capacity of common bean leaves to recover from photoinhibition under high-light intensity conditions could be genetically improved to achieve efficient photosynthesis and increase crop productivity.

## Materials and methods

### Plant materials and growth conditions

Dalong1 seeds were exposed to ^60^Co radiation at a dosage of 200 Gy to construct a mutant library of common bean. M3 generation plants were gown in the field in Pingfang District, Harbin City, Heilongjiang Province (45°70′N, 126°64′E). The seedling-lethal phenotype of the *pvsl1* mutant was observed. To map the causal gene for *pvsl1*, we constructed segregation populations by crossing PI60234 with wild-type M138 plants potentially carrying heterozygous *pvsl1*. These F2 segregation populations were grown in the field in Pingfang District, Harbin City, Heilongjiang Province. For morphological observation, the *pvsl1* mutant and wild-type were grown in incubators with light intensities of 100, 75, and 25 μmol·m^−2^·s^−1^ (16 h light/8 h dark) as well as in a greenhouse with supplemental LED light (from 3:00 to 19:00) with a light intensity of ~250 μmol·m^−2^·s^−1^. The cultivar PI60234 was generously provided by Dr. Dajun Liu from Heilongjiang University, China.

### INDEL marker development

The genomic DNAs of Dalong1 and PI60234 were extracted from leaf tissues of a single plant. Resequencing of genomic DNA was performed on an Illumina platform at Annoroad Gene Technology (Beijing, China). Illumina sequencing reads were mapped to the *Phaseolus vulgaris* v2.1 reference genome. For Dalong1, the total number of reads was 40,671,064, the number of HQ reads was 37,816,560, and the total number of bases was 6,100,659,600. For P16234, the total number of reads was 41,089,288, the number of HQ reads was 38,466,163, and the total number of bases was 6,163,393,200. Initially, the NGSQC Toolkit (version of v2.3.3) was used to filter out low-quality reads^21^. The reference sequence of *Phaseolus vulgaris* v2.1 was downloaded from https://phytozome-next.jgi.doe.gov/info/Pvulgaris_v2_1 with a Phytozome ID of 442 and NCBI taxonomy ID of 3885. Then, clean reads were analyzed using SpeedSeq software^22^. Through LUMPY software built into SeedSeq, the structural variants between P16234 and Dalong1 were called. The authenticity of those variants was manually checked using IGV version 2.5.0^23^.

### Map-based cloning

Mapping of the *PvSL1* locus was conducted using an F2 population segregating for *pvsl1*. DNA extraction from every individual leaf was carried out using the CTAB method^33^. Using the 124 INDEL markers, the *PvSL1* locus was initially mapped to a genomic region between PvM36 and PvM37 on chromosome 2. Three additional INDEL markers were developed according to polymorphisms between PvM36 and PvM37. To validate the candidate gene for the *PvSL1* locus, we manually examined all possible genetic variations that occurred in the delimited region as well as the CDSs of the 200 genes on either side of the delimited region between the *pvsl1* mutant and Dalong1 on IGV^23^.

### Subcellular localization

The CDSs of *PvSL1* and *pvsl1* were cloned from Dalong1 and *pvsl1* mutants and fused in frame into the expression vector p35S-GFP. The constructs were transformed into Agrobacterium and then coinfiltrated into *Nicotiana benthamiana* leaves. The p35S-GFP empty vectors were used as controls. Tobacco was kept under long-day (16 L:8D) conditions at 22 °C for at least 48 h. Leaves were observed under a laser confocal microscope.

### RNA extraction and expression analysis

For RT-qPCR analysis, the total RNA of leaves was extracted using an OminiPlantRNA Kit (CW25985, CEBIO). The quantity was measured by a Nanodrop 2000C (Thermo Fisher Scientific). An amount equal to 500 ng of the total RNA was used for reverse transcription by TransScript One-Step gDNA Removal and cDNA Synthesis SuperMix (AT311-03, TransGen Biotech). TransStart Top Green qPCR SuperMix (AQ131-04; TransGen Biotech) was used for the RT-qPCR assays. RT-qPCR was conducted using a LightCycler 96 (Roche). The reference gene of *PvTIP41* (*Phvul.007G041700.1*) was used as an internal control. Primer sequences are listed in Table S7. The 2^−ΔCT^ method was used to calculate the relative expression levels based on three technical replicates.

### Multiple-sequence alignment and phylogenetic analysis

There are 12 members of the FtsH family in Arabidopsis. We searched for homologous FtsH family proteins against the *Phaseolus vulgaris* genome (https://phytozome-next.jgi.doe.gov/info/Pvulgaris_v2_1). All sequences were verified to contain AAA-ATPase and protease (zinc binding site) domains using the SMART package (http://labix.org/smart^34^). Alignment of all members of FtsH of Arabidopsis and *Phaseolus vulgaris* protein sequences was performed using MEGA 5.0 software (www.megasoftware.net^35^). The neighbor-joining method was used to construct trees.

### Chlorophyll measurements

Chlorophyll content was measured as previously described^36^. A 0.2 g fresh leaf sample of each indicated plant was ground into powder in liquid nitrogen and mixed thoroughly with 20 mL of 80% acetone. The mixture stood at −20 °C for 24 h in darkness while being mixed several times. One milliliter of supernatant was measured for absorbance at 665 and 649 nm. Chlorophyll content was calculated using the following formulas:

Concentration of total chlorophyll = (20.2A_645_ + 8.02A_663_) mg/g

### Chlorophyll fluorescence analyses

F_V_/F_M_ was measured using an OS5p + Pulse Modulated Chlorophyll Fluorometer (OPTI-SCIENCES) following the manufacturer’s instructions. Plants were placed in darkness for 30 min before measurement.

### Immunoblot analysis

Intact chloroplasts were prepared according to a previous protocol^37^. Briefly, chloroplasts were isolated from leaf tissues (5 g) by gently grinding the materials in 50 ml extraction buffer (50 mM HEPES-KOH, pH 7.6; 0.33 M sorbitol; 2 mM Na_2_EDTA; 1 mM MgCl_2_; 1 mM MnCl_2_; 2 mM NaNO_3_; 1 mM NaH_2_PO_4_; and 2 mM sodium isoascorbate) on ice. To obtain intact thylakoids, isolated chloroplasts were lysed osmotically by suspending the chloroplast pellets in PBS (pH 7.5) for 4 h at 4 °C using a Rotational Mixer WH-986. Thylakoids were pelleted by centrifuging at 5000 × *g* for 5 min. The loading quantity was adjusted equally according to the D2 protein content since D2 protein was used as the control. Total proteins of thylakoid membranes were separated by denaturing SDS-PAGE and blotted for 1 h on PVDF. Blots were blocked immediately following transfer in 2% blocking reagent in 20 mM Tris and 137 mM sodium chloride at pH 7.6 with 0.1% (v/v) Tween-20 (TBS-T) for 1 h at room temperature with agitation. Blots were incubated with antibodies against the D1 and D2 proteins, AS05084 and AS06146 (https://www.agrisera.com), at a dilution of 1:50,000 for 1 h at room temperature with agitation. The antibody solution was decanted, and the blot was rinsed briefly twice and then washed once for 15 min and three times for 5 min in TBS-T at room temperature with agitation. Blots were incubated in secondary antibody (CW0103S, goat anti-rabbit IgG, HRP conjugated, CoWin Biosciences) diluted to 1:8000 for 1 h at room temperature with agitation. The blots were washed as mentioned above and developed for 5 min with SuperSignal Western Femto Maximum Sensitivity Substrate (34095, Thermo Fisher). Images of the blots were obtained using a CCD imager (Tanon-5200, Tanon Science & Technology).

### Transcriptome analysis

The leaves of Dalong1 and *pvsl1* mutant plants were sampled 10 days after germination in the field. RNA was sequenced on an Illumina HiSeq instrument at Annoroad Gene Technology (Beijing, China). Illumina sequencing reads were mapped to the *Phaseolus vulgaris v2.1* reference genome (https://phytozome-next.jgi.doe.gov/info/Pvulgaris_v2_1) using the protocol described at https://github.com/XSLiuLab/RNAseq-workflow.

### Statistical analysis

All experiments in this study were carried out at least three times, and similar results were obtained. The figures show only a representative result. Data in all bar graphs represent the mean ± SD. All statistical analyses were determined using Statistical Package for Social Sciences (SPSS) version 20 software. For multiple comparisons, statistical significance at the 5% level was determined using Duncan’s multiple range tests.

## Supplementary information

Supplement figure and table
