# Supplementary material for: A critical role of PvFtsH2 in the degradation of photodamaged D1 protein in common bean
Source: Hortic Res. 2021 Jun 1;8:126. doi: 10.1038/s41438-021-00554-7 (PMC8167180; doi:10.1038/s41438-021-00554-7)
Supplement: Supplementary file 1 — Supplement figure and table [file 41438_2021_554_MOESM1_ESM.docx]

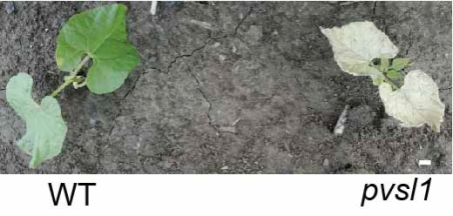


Supplement figure 1. *pvsl1* mutant died about two weeks after germination under national sunlight field condition.


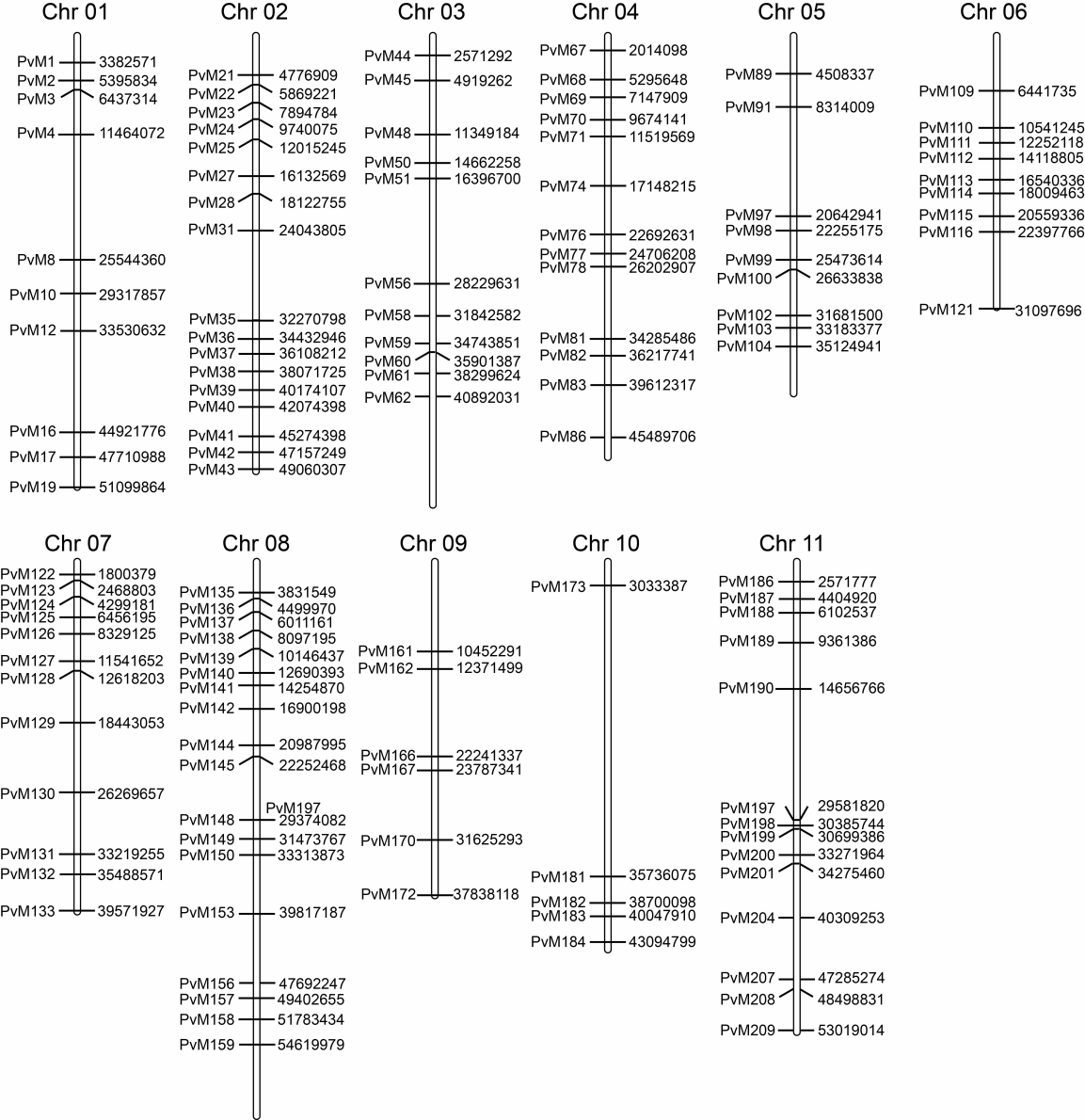


Supplement figure 2. Physical positions of 124 INsertion/DELetion markers in the common bean genome. Marker names are indicated on the left side and physical positions are presented on the right side.


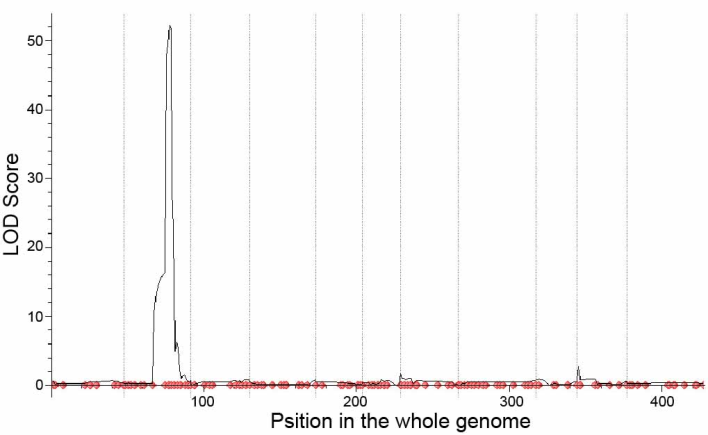


Supplement figure 3. QTL mapping of PvSL1locus in the F2 population. Using 124 INDEL markers we detected one candidate region (a genomic region between PvM36 and PvM37 on chromosome 2) for *PvSL1* locus by QTL IciMapping_4.0 software in F2 population.


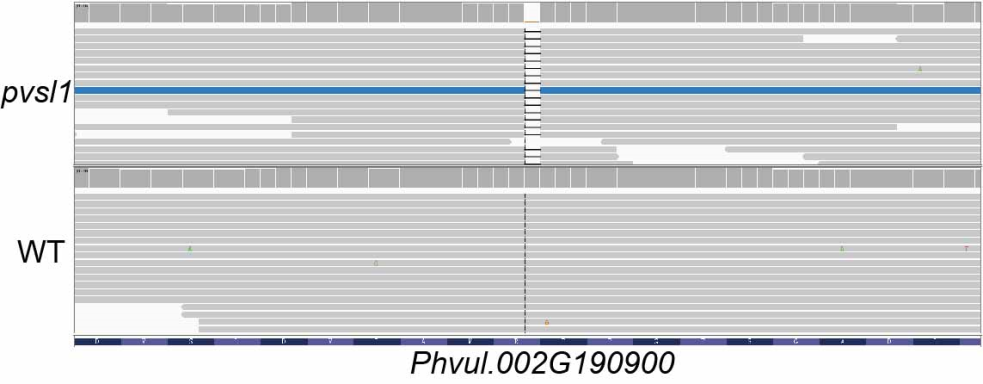


Supplement figure 4. A single base missing in CDS of *Phvul.002G190900* in pvsl1 mutant.


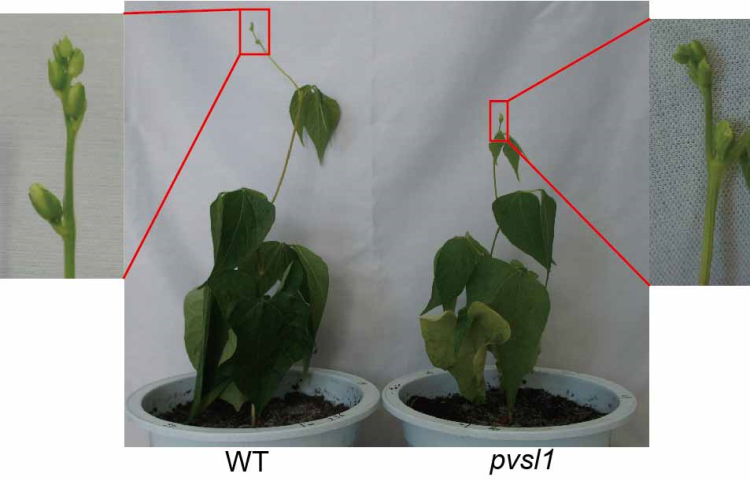


Supplement figure 5. *pvsl1* mutant flowered growing in an incubator with 75 μmol·m^-2^·s^-1^ light intensity.


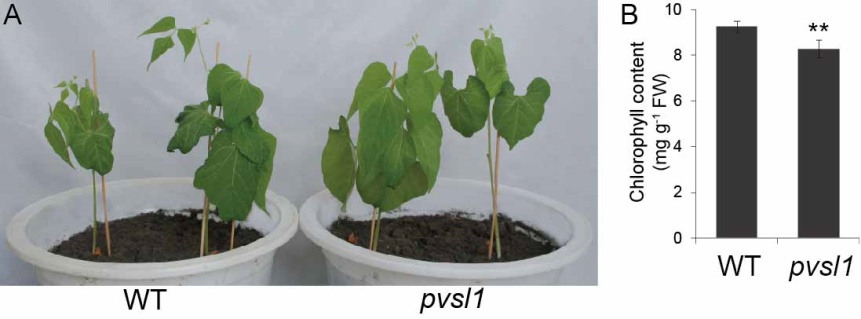


Supplement figure 6. A, Growing under 25 μmol·m^-2^·s^-1^, *pvsl1* mutant displayed similar leaf color phenotype with WT. B, The chlorophyll content of *pvsl1* was significantly decreased, although, it was close to that of WT.

Supplement table 1. Segregation patterns, Chi-square values and P-values for the F2 populations, which segregated seedling lethal plants.

| Total number of plants | No. of normal plants | No. of seedling lethal plants | Expected ratio | χ^2^ |
| --- | --- | --- | --- | --- |
| 425 | 320 | 105 | 3:1 | 0.889 |

Chi-square values calculated to test goodness of fit to a 3:1 ratio; *P* = probability of a greater value of chi-square.

Supplement table 2. INDEL markers for genetic mapping based in common bean.

| Chromosome | Marker name | Marker position | Forward primer | Reverse primer |
| --- | --- | --- | --- | --- |
| Chr 01 | PvM1 | 3382571 | TGGAAGAATCAATCCCCATC | TGTAAACCGCGAGACATTAAAA |
|  | PvM2 | 5395834 | GCTAGGATAAGCACAATTTTCCA | AAGTGGGTGCAAACCTCAAC |
|  | PvM3 | 6437314 | TGTCCCACATTAGTCAGGACTCT | AAAACTTAATAACCACCGAAACACA |
|  | PvM4 | 11461072 | TGCATAAAGATTCCCATACAAGA | AAAGCCCTATTAACATGAAAGCA |
|  | PvM8 | 25544360 | AGCAGGAGAAACTATAACACTGGA | AGGATGACTAAATATCCCAACCT |
|  | PvM10 | 29317857 | TGATCTTCAATCGTTTGTTTCA | GGCACTTTCACCATTCCTTG |
|  | PvM12 | 33530632 | CGCTGAAACCAAAACGAGAC | TTTCCATCGAAGTTGGGACT |
|  | PvM16 | 44921776 | TGGTTTACAGGGAGTGCTGTT | TGAAGGTGGCACAAGAAGAA |
|  | PvM17 | 47710988 | CAACTGGGCCAAGTAGTTTCA | TGCATCAATGTTCAGAATCG |
|  | PvM19 | 51099864 | CGACGAAGCGCACACTATTA | CACCACTTCTAACCAATTTCCA |
| Chr 02 | PvM21 | 4776909 | GGGTTGAGTTAGGACAATGAAAG | TCCTCAGCATTTCATGCAGTA |
|  | PvM22 | 5869221 | TCTGCATGTGCATGTGTGTT | AACAATGAGATGGCTGCACA |
|  | PvM23 | 7894784 | CCCATCACTCACTTTGCTCA | TGCTTCCTTCCGTGAGTCTT |
|  | PvM24 | 9740075 | CACAGTTGCTTTGGATGGTC | AGAGCTGAGTGTGTGCAAGC |
|  | PvM25 | 12015245 | ATGGGCCTAATGATGTGTCA | GACATTCCTTCAACAATTTCATCA |
|  | PvM27 | 16132569 | GACTCTTCACCAGCAGATCG | TTTCCAGGTTTGAGGACTCG |
|  | PvM28 | 18122755 | ACCGAACGAGCTTTTGATTG | TCAGCTTTGATTCTTGCTTCC |
|  | PvM31 | 24043805 | CATTTCTGTTAATATGCTCGAGTGA | CCATTGATAACAACCACTTTT |
|  | PvM35 | 32270798 | TTACAACGCAACATCAGTCG | GTTGATGGTGTATCGCATGG |
|  | PvM36 | 34432946 | TGGATGGAAGCATGAAATTG | TTGTTTTCTTCATTGAGGCATTT |
|  | PvM37 | 36108212 | CAAGTTCTGAAGGGCGGTTA | CAGATGTTAGGAGGACCCAAG |
|  | PvM38 | 38071725 | TGGACTCCTCCTTGAGCAAT | CTCCACAAGCATATCAAACACC |
|  | PvM39 | 40174107 | CAGGTATAGTGAAGTGGTACGTGAA | TGAGGCTCAAGGTCATACAT |
|  | PvM40 | 42074398 | TGAAACTCCTTTGTGTTTCTCG | TTCTCCCTGTTTTCCGCATA |
|  | PvM41 | 45274398 | CAATTAGGCTAACCAATCTACGC | CCAAGTTCAATCATCCAAACAA |
|  | PvM42 | 47157249 | TCCAACATCTCTAATGCGAAA | TTTGTCTCTCCTTGCACTGG |
|  | PvM43 | 49060307 | TTCTGCAAATCAACGCTCTG | AAGTTTTAATGCAAGCTTAGACCA |
| Chr 03 | PvM44 | 2571292 | CCTGCACAAGAAGACATCCA | TGCTTTTAGAGAGCGGTTTTT |
|  | PvM45 | 4919262 | CAATCCCAACACTTGTTCTA | CGATTTTTCTATCAATGTTTGTTGA |
|  | PvM48 | 11349184 | TGGTCAGTGAAGGAACATGG | TTTTTGGAGCCAACTATTCC |
|  | PvM50 | 14662258 | ATACCAACCTCCACCGAACC | GCATGCATGACAGCTGTAGACT |
|  | PvM51 | 16396700 | CCCTGTAATTGTTTTGTGGGATA | CGGACATATCTTGCTCCACTT |
|  | PvM56 | 28229631 | CCTTGAGGGTTTCAGTTGGA | GACTATAGCCATCCCCCACA |
|  | PvM58 | 31842582 | ACAAGCCGACCCACGTATTA | ACCTAGGATCGGACCAACG |
|  | PvM59 | 34743851 | TTGGATGGGTAATCTTTTAGGG | ACCATCAACCACAATGCAAA |
|  | PvM60 | 35901387 | TGTGAACCATGATTCTTTTTGG | GCAAACCTTGGCTACATCG |
|  | PvM61 | 38299624 | AGGTTGCACGTGAAGTCATC | CCACTACGAATCTTAATACCAAAAA |
|  | PvM62 | 40892031 | GATCGGTCTCTGAAGGTTGG | CGAGTGCTATCTTCTCTCTCGTT |
| Chr 04 | PvM67 | 2014098 | TTGATTTGTCTCAAGCTCAACC | TTCATGTCCATGTTAGCCTCA |
|  | PvM68 | 5295648 | TTTTCATCCATGATCCTAGTCG | AACAATGGTAAGTGAAATCAAGCA |
|  | PvM69 | 7147909 | TCTTCGCTCCCTCATTCCTA | CCGAAAATATTGATTTCTTGGA |
|  | PvM70 | 9674141 | TCTCGATAGGCTTTCCCTCA | GGAGTGCACAATGCCTTTCT |
|  | PvM71 | 11519569 | CGCATCGTTTACTGCTGTTG | CTGGCGAGATCAGTGTGAAA |
|  | PvM74 | 17148215 | GGATTGCCGAGATAAGGTGA | CCCCTAACTTCATGGGATGT |
|  | PvM76 | 22692631 | TCAACAAGGCACGCTAAGTG | TTGATCTCCATCAAGTTTGGAA |
|  | PvM77 | 24706208 | CCCAAGTCATCTTAGGGAAGTC | AAGCTGGCTAAACCATGGAA |
|  | PvM78 | 26202907 | TGGTTTAGGGTTGTGGTTGAA | CATGAAGTGCCTAACATCAACA |
|  | PvM81 | 34285486 | TCTGCTGAAATCCCTTTGCT | CAGCAATCAAACTCAAAGGAG |
|  | PvM82 | 36217741 | AACCCCATGATGGTCAAAAA | TCCAATGCAGAATCACAGAGA |
|  | PvM83 | 39612317 | TGGTTACATCCATTATGAAGCAG | CAATCAGTATAATGAAGGGGAACA |
|  | PvM86 | 45489706 | TAACCGGATGACCGATTACC | CAACCATTACCTTTCACCATGA |
| Chr 05 | PvM89 | 4508337 | TCAGGGGAGGAAACTTGTTG | GAATGGCGAATCTTCCTCAA |
|  | PvM91 | 8314009 | AAAAAGTGACCAGTGAAACTTAAA | TCCTTCCCATTATACAAATCGAG |
|  | PvM97 | 20642941 | AATCAAACAACAACCCTACAA | TTGGCTTTGTTAATGCTGCTT |
|  | PvM98 | 22255175 | CGTTGATCCATTCGATTTAACC | TCGAGATTCTCCGGATAAAAG |
|  | PvM99 | 25473614 | ACAGTATCTGGTCGCCGTTG | TGGTGGTAGGGTCAAAGCAT |
|  | PvM100 | 26633838 | GGAAGGTACATGGCGTTTTC | TGGGAAATGAAGGTCAATCA |
|  | PvM102 | 31681500 | TGGGCATTTTGGTATTTTCA | TCCCAGTTCCATGACACTTTC |
|  | PvM103 | 33183377 | GCCTTCTGACTGGACGTGTT | TGCATCCATATCATCCCCTTA |
|  | PvM104 | 35124941 | CTCGGAAATCTCCAGGAAGT | TCCAGTAATAACAGGCCAAGC |
| Chr 06 | PvM109 | 6441735 | GCGAAGTCCTTTAAATAGACACAAA | CCATGACAAAAACGGATGTG |
|  | PvM110 | 10541245 | TGTTCACAGGCAAAATGAAGA | CCATGATCCAACCCAGGTAA |
|  | PvM111 | 12252118 | GGACATTCCACGTCCTCAGT | GGATCTACGCACGGTACCTC |
|  | PvM112 | 14118805 | GGGTACGGTAGGACGACAGA | TCAAAATGGTTTAGCTTTTTCAT |
|  | PvM113 | 16540336 | GCCTAACAGACCGATCCAAA | AAATTGGCAAATTGGGCTTA |
|  | PvM114 | 18009463 | TTGTTTTGTGATGACCAGGA | TCCTTTGATAAAAGATTACATTTCG |
|  | PvM115 | 20559336 | ATTCTGTTGTCGGGTGAAGC | AACATATTCCAATCGGCTACT |
|  | PvM116 | 22397766 | GAAGGTTCACGTGGAGGAAA | TTGAAAGCACTGGTCTCTGG |
|  | PvM121 | 31097696 | TCGGTAAAATGTCAAATTCAAAAA | CAGGGTTAGCCTTTTTCACC |
| Chr 07 | PvM122 | 1800379 | GGTGCAAACAGACAAATCCA | TCCTCACTCCTCTGCTGCAT |
|  | PvM123 | 2468803 | TCACAAGCAGGTAACGTGATTTA | TGAAGAGGATCTCCACAAAGA |
|  | PvM124 | 4299181 | AAAATGACTCATCCTAAAAGCTGAA | TCCTCAGTCTATTTAGACCCCAAC |
|  | PvM125 | 6456195 | TTGTCGACTTGGTCCGATCT | CGACATTTCTACGGGTGGAC |
|  | PvM126 | 8329125 | TGTCCTATATAAATAAAAATCTGAAA | TTACGCAGTGTTTGCTTTCG |
|  | PvM127 | 11541652 | GGTGCTTGAACATACAGTGGA | TGAATTCCTTTCACCGCTTC |
|  | PvM128 | 12618203 | CGTGAAATCAATGCAATAGTCA | GATCAAAGAGGCAGCAGACC |
|  | PvM129 | 18443053 | CCTTCCTCCCTCACTTCCTC | GGACACCTCATGACTCAGCA |
|  | PvM130 | 26269657 | CGTGATGGCTCAACATAAGAG | AAATGGGTGCAAACCTCATC |
|  | PvM131 | 33219255 | TCGGCGCATGTGTTTTTAT | TCCTGGAAACCAATTTCGAC |
|  | PvM132 | 35488571 | AAGCTTCACCATGATCTTTTCA | AAAATGATTGCTCTTCAACTGC |
|  | PvM133 | 39571927 | CCATAAAACAGGACCCCAAA | CCATCAAAGAGCCCTGCTAA |
| Chr 08 | PvM135 | 3831549 | TGGGGAACAAATTCTCTTACAAA | TCAACTGAGTTTCTGAACCGTAA |
|  | PvM136 | 4499970 | TTGGTGTAGGATGCAAAAGG | ACGAGCTAGAACCGCTCAAA |
|  | PvM137 | 6011161 | GCAAAGGGTGCTTAAAGTGC | CCAACCCAAACTCATTTCTCA |
|  | PvM138 | 8097195 | AGTTTGTCACATTGTTACCTGAAG | GATGTCATACTTGGGGCACA |
|  | PvM139 | 10146437 | GCCTAGGCCCATTTCTATGT | AACCGGATAAGGAGACACACA |
|  | PvM140 | 12690393 | GAGAAGCAGGAGGACGAGAA | TGGTGTGGTTGTTATTTCTTCAA |
|  | PvM141 | 14254870 | GGTGCAAACACAGAGAGAAACA | ATTGATTCGCAAAACCTCCA |
|  | PvM142 | 16900198 | GGATTTGAAGGAAAGATTCTCCA | TTCTATGAGCCCCTCTCCATT |
|  | PvM144 | 20987995 | AACATGTTTAAACTCTACCAAAAA | GTTTGCGGATCAATTTGGTT |
|  | PvM145 | 22252468 | ATGTAACCCACAATCCCAAT | TCTCCTCAAATTCGTGACAAAA |
|  | PvM148 | 29374082 | AGCCGGTCTCTTTCACAATG | GAATTTCCAATCGATCTTCATCA |
|  | PvM149 | 31473767 | AGAGAAAACCCACGCAAATG | TTGAAAATCCAAAGCCTGATG |
|  | PvM150 | 33313873 | AAGGACAACCCATGCAAAAG | GAAGCATTCAAAGAGGAATTGA |
|  | PvM153 | 39817187 | TTGTGATGCACACACACTTCTT | CTACGGGGAGGTAAACACCA |
|  | PvM156 | 47692247 | TGGCAGGCAACTATGTACCA | AAAGGCAAACATCTTCTTTTGG |
|  | PvM157 | 49402655 | TGTCACTGTATGCGTCACGTT | AAATGTCCCACTTGCCATTC |
|  | PvM158 | 51783434 | CAGGGGAAAACTTTAGAACCAA | TTGTTCTCTTTTCCCTTTTGC |
|  | PvM159 | 54619979 | AACACAACTATTTGCCCTTTCAA | GCTCAATCACAGTTTTTGTTATGG |
| Chr 09 | PvM161 | 10452291 | TTGTGAACGTCCAACTGCAT | TTGTATCACACTCCCTGTGGA |
|  | PvM162 | 12371499 | CCACAAACCAAATTCAAGCA | GAATGAAGAGGGGCATCCAT |
|  | PvM166 | 22241337 | AAGCAAACCAGTCAGACTAGCA | ATTGCTCACATTGGTGGTGA |
|  | PvM167 | 23787341 | AGTGCATGGAGTAGGCAGGT | CGCACACCCTAACACACACT |
|  | PvM170 | 31625293 | AAGACTTGGCTTAATTCGATCTG | GGAAAACCGAAGCAATAAAAA |
|  | PvM172 | 37838118 | GACCAACATACTTCATCCCATGT | TCAAGGATTCAGTTTCCATGC |
| Chr 10 | PvM173 | 3033387 | GAATCTTTGCAGTGAAGGTTTGAATG | GATTTCAGAAATTTGAACTTGAAGGTC |
|  | PvM181 | 35736075 | CCATTCTTAGTTTCTCACCTGTGG | GAATTAGACAGAGCCATAAGTC |
|  | PvM182 | 38700098 | GTGGTGAGTAAAGATCTAGAACG | TTTTATGGGATCGGATAAGATTC |
|  | PvM183 | 40047910 | GCAGAAGCAGTTCATCATATGTTC | CCATCCAAATTCTCATCACACC |
|  | PvM184 | 43094799 | CGTTATCCAAATTCCCGTTATTGG | TGAAAGTTTAGAGATTAACTAACC |
| Chr 11 | PvM186 | 2571777 | TTTGTGGCACACAGACACAG | TCATTTCGATAGAAACATTTGGAA |
|  | PvM187 | 4404920 | AACAGGAGATTCCAAAAGGGTA | TTTTGAATTTCGTAATAAGAATTAGGC |
|  | PvM188 | 6102537 | TTGATGCGAACATACCTAGCA | GTTGGCGGTGAAGTGAGAC |
|  | PvM189 | 9361386 | CCATAAAAGAGAAGACGGAAAA | CAATCTTGTGCAATTACGTTCAA |
|  | PvM190 | 14656766 | TGTGATGCTAATAGGGGTTTCA | AAAAAGTGTGTAAATGATGGTATGC |
|  | PvM197 | 29581820 | AATATCGGCCTATTCTGTTGG | TGTGCAACGGTAAAAGATGG |
|  | PvM198 | 30385744 | GACTTTTGACCCACGCAAAG | CAGACTAGCCGACGCAAAAC |
|  | PvM199 | 30699386 | CCAGGAGTCAATCAAGAACAGA | TGAACAAGAGCATTTTATTCCAAA |
|  | PvM200 | 33271964 | TTCCCAGTCTTAGGGCATTC | GATGAAGGGTGCAAGAGCAT |
|  | PvM201 | 34275460 | GGACAAAGCTCGAGATCTTTTATT | TTCCGTTTACTTCCTTCATTAACC |
|  | PvM204 | 40309253 | GTTGGCCAGTTTTCATTCGT | TCTTTCATCCTGGTTGTCCA |
|  | PvM207 | 47285274 | TGGAATTTTTGCAGAAGTGG | AATTCCCAATCTGGAAGCCTA |
|  | PvM208 | 48498831 | ACCGACTTGAGGTCGAAACA | CTGACAAAGTGTTAGTTGGAGTGA |
|  | PvM209 | 53019014 | TGAAGTCTATATTCCACCACCT | GGATTTTTACTTGGGTTTGGTTT |

Supplement table 3. QTLs for PvSL1 were mapped in the F2 population.

| Trait name | Chromosome | Left marker | Right marker | LOD | PVE(%) |
| --- | --- | --- | --- | --- | --- |
| PvSL1 | 2 | PvM36 | PvM37 | 52.2306 | 86.4846 |
| PvSL1 | 2 | PvM38 | PvM39 | 6.2057 | 0.8796 |
| PvSL1 | 10 | PvM174 | PvM259 | 2.6914 | 0.4981 |

PvSL1 locus was mapped in the region between PvM36 and PvM37 using F2 population.

Supplement table 4. The INDEL markers were used to fine map PvSL1locus.

| Marker name | Marker position | Forward primer | Reverse primer |
| --- | --- | --- | --- |
| PvM355 | 35394272 | TGGCTCAAATAGCTTCCATTTCG | CTTTCCCTTCGAAACATTCCAAG |
| PvM357 | 35448003 | GAATAAACCCCACGCACCATG | CCAATTTCATGCAGTGAGAAATGC |
| PvM343 | 35546520 | AGACTTAGATATCAGGTGGTG | CATCGCAGTACACAACAAAGC |

Supplement table 5. Similarities of FtsH2, FtsH8, PvSL1 and Phvul.009G241100

| Similarity | FtsH2 | FtsH8 | PvSL1 | Phvul.009G241100 |
| --- | --- | --- | --- | --- |
| FtsH2 | ■ | 88.98% | 84.11% | 82.34% |
| FtsH8 | 88.98% | ■ | 83.62% | 83.83% |
| PvSL1 | 84.11% | 83.62% | ■ | 89.53% |
| Phvul.009G241100 | 82.34% | 83.83% | 89.53% | ■ |

Supplement table 6. Some of GO biological processes associated with *pvsl1* mutant differentially expressed genes.

| Biological processes | *pvsl1*/WT | |
| --- | --- | --- |
|  | Up-regulated | Down-regulated |
| Regulation of primary metabolic process | 185 (8.2%) | 57 (2.9%) |
| Photosynthesis | 31 (1.4%) | 0 |
| Programmed cell death | 33 (1.5%) | 0 |
| Homeostatic process | 16 (0.7%) | 17 (0.9%) |
| Protein ubiquitination | 58 (2.6%) | 20 (1.0%) |
| Ribosomal protein | 3 (0%) | 198 (10.3%) |
| Others | 1930 (85.6%) | 1668 (84.9%) |
| Total no. of terms | 2253 | 1964 |

Supplement table 7. Different expression genes associated with ribosomal in pvsl1 mutant.

| Gene ID | WT | WT | *pvsl1* | *pvsl1* | Best-hit Arabidopsis name | Arabidopsis definition |
| --- | --- | --- | --- | --- | --- | --- |
| Phvul.001G003200 | 34881 | 44569 | 11420 | 10695 | AT5G39740.1 | ribosomal protein L5 B |
| Phvul.001G007700 | 20104 | 22891 | 9386 | 8299 | AT2G18110.1 | Translation elongation factor EF1B/ribosomal protein S6 family protein |
| Phvul.001G008500 | 5394 | 4461 | 1136 | 1086 | AT5G24510.1 | 60S acidic ribosomal protein family |
| Phvul.001G017500 | 51913 | 50369 | 47898 | 47759 | AT1G74970.1 | ribosomal protein S9 |
| Phvul.001G058350 | 499 | 292 | 100 | 134 | ATCG01120.1 | chloroplast ribosomal protein S15 |
| Phvul.001G079100 | 37538 | 23885 | 6818 | 6220 | AT3G61110.1 | ribosomal protein S27 |
| Phvul.001G104800 | 6123 | 8926 | 2254 | 2197 | AT1G80750.1 | Ribosomal protein L30/L7 family protein |
| Phvul.001G137900 | 661 | 2661 | 463 | 475 | AT3G15460.1 | Ribosomal RNA processing Brix domain protein |
| Phvul.001G139900 | 32661 | 24601 | 7536 | 7114 | AT3G47370.3 | Ribosomal protein S10p/S20e family protein |
| Phvul.001G147600 | 1016 | 2228 | 581 | 494 | AT1G03360.1 | ribosomal RNA processing 4 |
| Phvul.001G174900 | 10126 | 10327 | 2941 | 2656 | AT5G02960.1 | Ribosomal protein S12/S23 family protein |
| Phvul.001G175000 | 10854 | 4085 | 1043 | 952 | AT5G02960.1 | Ribosomal protein S12/S23 family protein |
| Phvul.001G184700 | 42803 | 27757 | 10417 | 9072 | AT2G36620.1 | ribosomal protein L24 |
| Phvul.001G188201 | 43762 | 26386 | 9150 | 8029 | AT3G44590.2 | 60S acidic ribosomal protein family |
| Phvul.001G204100 | 42604 | 46231 | 12218 | 11362 | AT3G16780.1 | Ribosomal protein L19e family protein |
| Phvul.001G204300 | 42052 | 37990 | 13074 | 11470 | AT3G16780.1 | Ribosomal protein L19e family protein |
| Phvul.001G208200 | 16537 | 14356 | 3667 | 3347 | AT3G05590.1 | ribosomal protein L18 |
| Phvul.001G235900 | 43280 | 25926 | 8067 | 7134 | AT1G52300.1 | Zinc-binding ribosomal protein family protein |
| Phvul.001G244200 | 31994 | 44108 | 11399 | 10558 | AT1G08360.1 | Ribosomal protein L1p/L10e family |
| Phvul.002G048000 | 13060 | 12307 | 3550 | 3702 | AT1G07770.2 | ribosomal protein S15A |
| Phvul.002G051100 | 19570 | 13477 | 3057 | 2663 | AT3G47370.3 | Ribosomal protein S10p/S20e family protein |
| Phvul.002G052200 | 16395 | 11300 | 2815 | 2651 | AT3G43980.1 | Ribosomal protein S14p/S29e family protein |
| Phvul.002G057000 | 93372 | 68584 | 16638 | 15776 | AT4G16720.1 | Ribosomal protein L23/L15e family protein |
| Phvul.002G063300 | 27285 | 17166 | 5776 | 5284 | AT5G56670.1 | Ribosomal protein S30 family protein |
| Phvul.002G074600 | 7254 | 13933 | 3580 | 3185 | AT2G40010.1 | Ribosomal protein L10 family protein |
| Phvul.002G085600 | 23825 | 22213 | 7965 | 6850 | AT1G26880.1 | Ribosomal protein L34e superfamily protein |
| Phvul.002G150100 | 38833 | 40497 | 16433 | 14757 | AT1G70600.1 | Ribosomal protein L18e/L15 superfamily protein |
| Phvul.002G174200 | 43575 | 34841 | 14161 | 11986 | AT1G67430.1 | Ribosomal protein L22p/L17e family protein |
| Phvul.002G177700 | 21306 | 16896 | 6589 | 5468 | AT3G59540.1 | Ribosomal L38e protein family |
| Phvul.002G182800 | 2965 | 6084 | 1822 | 1575 | AT3G18240.1 | Ribosomal protein S24/S35, mitochondrial |
| Phvul.002G190100 | 35919 | 31982 | 5656 | 5312 | AT4G15000.1 | Ribosomal L27e protein family |
| Phvul.002G190400 | 48351 | 31508 | 9835 | 8879 | AT1G74270.1 | Ribosomal protein L35Ae family protein |
| Phvul.002G208200 | 4840 | 4120 | 1544 | 1366 | AT1G26740.1 | Ribosomal L32p protein family |
| Phvul.002G223200 | 16131 | 12021 | 4698 | 4303 | AT2G34480.1 | Ribosomal protein L18ae/LX family protein |
| Phvul.002G229900 | 23588 | 36048 | 7176 | 6752 | AT5G10360.1 | Ribosomal protein S6e |
| Phvul.002G233700 | 2874 | 3668 | 998 | 921 | AT4G30930.1 | Ribosomal protein L21 |
| Phvul.002G236400 | 15870 | 13940 | 2667 | 2421 | AT4G18100.1 | Ribosomal protein L32e |
| Phvul.002G254000 | 26116 | 31196 | 8915 | 7944 | AT5G23740.1 | ribosomal protein S11-beta |
| Phvul.002G258200 | 5608 | 4020 | 916 | 873 | AT1G16790.1 | ribosomal protein-related |
| Phvul.002G259300 | 55569 | 41436 | 12103 | 11156 | AT4G25890.1 | 60S acidic ribosomal protein family |
| Phvul.002G265000 | 32098 | 31827 | 8637 | 8055 | AT1G74050.1 | Ribosomal protein L6 family protein |
| Phvul.002G305300 | 4300 | 4870 | 1709 | 1476 | AT4G29430.1 | ribosomal protein S15A E |
| Phvul.002G305600 | 19134 | 16432 | 4778 | 4096 | AT2G19740.1 | Ribosomal protein L31e family protein |
| Phvul.002G317200 | 42432 | 48962 | 12193 | 10914 | AT3G02560.1 | Ribosomal protein S7e family protein |
| Phvul.002G320200 | 49145 | 38695 | 11330 | 10352 | AT5G09510.1 | Ribosomal protein S19 family protein |
| Phvul.002G321400 | 11534 | 15833 | 3705 | 3578 | AT3G57490.1 | Ribosomal protein S5 family protein |
| Phvul.003G017600 | 3340 | 6379 | 2513 | 2284 | AT4G01560.1 | Ribosomal RNA processing Brix domain protein |
| Phvul.003G018300 | 71121 | 95081 | 26274 | 24968 | AT3G57490.1 | Ribosomal protein S5 family protein |
| Phvul.003G020600 | 57333 | 117504 | 40206 | 37695 | AT2G40010.1 | Ribosomal protein L10 family protein |
| Phvul.003G025200 | 24134 | 37673 | 10687 | 10077 | AT1G74060.1 | Ribosomal protein L6 family protein |
| Phvul.003G089200 | 33818 | 29359 | 9886 | 8676 | AT5G09510.1 | Ribosomal protein S19 family protein |
| Phvul.003G095800 | 22543 | 29165 | 6416 | 5790 | AT5G59850.1 | Ribosomal protein S8 family protein |
| Phvul.003G112200 | 15486 | 13900 | 4664 | 4304 | AT3G06680.1 | Ribosomal L29e protein family |
| Phvul.003G132300 | 6788 | 8055 | 2458 | 2231 | AT1G07770.2 | ribosomal protein S15A |
| Phvul.003G163950 | 11162 | 9568 | 3647 | 3309 | AT4G22380.1 | Ribosomal protein L7Ae/L30e/S12e/Gadd45 family protein |
| Phvul.003G173900 | 24146 | 23106 | 6098 | 5673 | AT5G23740.1 | ribosomal protein S11-beta |
| Phvul.003G191200 | 58842 | 57185 | 11554 | 10614 | AT5G39850.1 | Ribosomal protein S4 |
| Phvul.003G204100 | 36268 | 57992 | 14021 | 12894 | AT1G70600.1 | Ribosomal protein L18e/L15 superfamily protein |
| Phvul.003G214600 | 13031 | 15365 | 4402 | 4196 | AT4G00100.1 | ribosomal protein S13A |
| Phvul.003G220800 | 31950 | 19657 | 6730 | 5621 | AT5G56670.1 | Ribosomal protein S30 family protein |
| Phvul.003G222000 | 52741 | 36702 | 14036 | 12687 | AT4G16720.1 | Ribosomal protein L23/L15e family protein |
| Phvul.003G246600 | 4068 | 8570 | 2637 | 2242 | AT5G66860.1 | Ribosomal protein L25/Gln-tRNA synthetase, anti-codon-binding domain |
| Phvul.003G257500 | 1951 | 5799 | 1598 | 1391 | AT4G36420.1 | Ribosomal protein L12 family protein |
| Phvul.003G261000 | 29722 | 32161 | 9851 | 9080 | AT4G36130.1 | Ribosomal protein L2 family |
| Phvul.003G261200 | 37155 | 37092 | 9318 | 8396 | AT2G17360.1 | Ribosomal protein S4 (RPS4A) family protein |
| Phvul.003G262000 | 2980 | 2417 | 913 | 749 | AT5G14290.1 | Mitochondrial ribosomal protein L37 |
| Phvul.003G262900 | 22068 | 28872 | 7841 | 7306 | AT4G36130.1 | Ribosomal protein L2 family |
| Phvul.004G046700 | 28731 | 29919 | 5926 | 5857 | AT1G33140.1 | Ribosomal protein L6 family |
| Phvul.004G063700 | 20600 | 11990 | 3405 | 3269 | AT1G36240.1 | Ribosomal protein L7Ae/L30e/S12e/Gadd45 family protein |
| Phvul.004G065100 | 32045 | 34088 | 8809 | 7862 | AT5G02450.1 | Ribosomal protein L36e family protein |
| Phvul.004G065700 | 17944 | 13312 | 3243 | 2900 | AT3G02560.2 | Ribosomal protein S7e family protein |
| Phvul.004G131800 | 9679 | 9741 | 3157 | 2708 | AT5G35530.1 | Ribosomal protein S3 family protein |
| Phvul.004G131900 | 47212 | 44951 | 18177 | 15962 | AT2G31610.1 | Ribosomal protein S3 family protein |
| Phvul.004G153600 | 2182 | 2116 | 682 | 713 | AT3G18240.1 | Ribosomal protein S24/S35, mitochondrial |
| Phvul.004G156132 | 20622 | 21976 | 8516 | 7932 | AT3G02560.2 | Ribosomal protein S7e family protein |
| Phvul.004G172800 | 33801 | 32393 | 10628 | 9049 | AT2G19740.1 | Ribosomal protein L31e family protein |
| Phvul.005G011400 | 35492 | 27676 | 11028 | 9579 | AT3G58700.1 | Ribosomal L5P family protein |
| Phvul.005G012100 | 3242 | 6253 | 1277 | 1202 | AT3G17465.1 | ribosomal protein L3 plastid |
| Phvul.005G016800 | 2878 | 4018 | 1275 | 1090 | AT5G64670.1 | Ribosomal protein L18e/L15 superfamily protein |
| Phvul.005G018600 | 42538 | 32815 | 7610 | 6981 | AT3G02080.1 | Ribosomal protein S19e family protein |
| Phvul.005G031000 | 4424 | 2773 | 1031 | 961 | AT1G63780.1 | Ribosomal RNA processing Brix domain protein |
| Phvul.005G038900 | 16399 | 23762 | 5172 | 4585 | AT1G74060.1 | Ribosomal protein L6 family protein |
| Phvul.005G043400 | 15962 | 9201 | 3593 | 3308 | AT3G04770.2 | 40s ribosomal protein SA B |
| Phvul.005G055500 | 3036 | 3584 | 874 | 752 | AT3G58660.1 | Ribosomal protein L1p/L10e family |
| Phvul.005G107000 | 39762 | 38999 | 11416 | 10189 | AT2G37270.1 | ribosomal protein 5B |
| Phvul.005G123700 | 32616 | 46607 | 16337 | 14403 | AT1G08360.1 | Ribosomal protein L1p/L10e family |
| Phvul.005G126000 | 35201 | 29302 | 8775 | 8136 | AT3G44590.2 | 60S acidic ribosomal protein family |
| Phvul.005G126800 | 8028 | 7135 | 2558 | 2222 | AT2G44860.1 | Ribosomal protein L24e family protein |
| Phvul.005G154400 | 19576 | 9811 | 2384 | 2480 | AT3G59540.1 | Ribosomal L38e protein family |
| Phvul.005G161300 | 26826 | 25192 | 8474 | 7651 | AT2G19730.2 | Ribosomal L28e protein family |
| Phvul.005G182200 | 23446 | 34148 | 8903 | 7871 | AT2G32060.1 | Ribosomal protein L7Ae/L30e/S12e/Gadd45 family protein |
| Phvul.006G021600 | 1547 | 2302 | 842 | 927 | AT4G37660.1 | Ribosomal protein L12/ ATP-dependent Clp protease adaptor protein ClpS family protein |
| Phvul.006G055700 | 4476 | 6151 | 1675 | 1593 | AT4G11630.1 | Ribosomal protein L19 family protein |
| Phvul.006G060600 | 17351 | 17620 | 5635 | 5139 | AT5G23740.1 | ribosomal protein S11-beta |
| Phvul.006G060900 | 13274 | 12088 | 1893 | 1950 | AT5G08180.1 | Ribosomal protein L7Ae/L30e/S12e/Gadd45 family protein |
| Phvul.006G088800 | 31251 | 29242 | 8256 | 7097 | AT3G05590.1 | ribosomal protein L18 |
| Phvul.006G089100 | 19147 | 12722 | 4378 | 4005 | AT3G05560.3 | Ribosomal L22e protein family |
| Phvul.006G099500 | 37989 | 36172 | 8117 | 7504 | AT5G28060.1 | Ribosomal protein S24e family protein |
| Phvul.006G124100 | 2485 | 3332 | 1329 | 1092 | AT3G06040.3 | Ribosomal protein L12/ ATP-dependent Clp protease adaptor protein ClpS family protein |
| Phvul.006G128500 | 35470 | 30582 | 9622 | 8377 | AT5G48760.1 | Ribosomal protein L13 family protein |
| Phvul.006G146000 | 2052 | 2692 | 722 | 761 | AT2G20060.1 | Ribosomal protein L4/L1 family |
| Phvul.006G155200 | 35482 | 40565 | 13945 | 12470 | AT1G07770.2 | ribosomal protein S15A |
| Phvul.006G163400 | 77761 | 72559 | 20981 | 20290 | AT2G34480.1 | Ribosomal protein L18ae/LX family protein |
| Phvul.006G163500 | 35070 | 22762 | 7949 | 6865 | AT3G52580.1 | Ribosomal protein S11 family protein |
| Phvul.006G163600 | 16849 | 15084 | 4348 | 3892 | AT3G52580.1 | Ribosomal protein S11 family protein |
| Phvul.006G168118 | 11256 | 8018 | 2782 | 2324 | AT3G59540.1 | Ribosomal L38e protein family |
| Phvul.006G180400 | 2465 | 3939 | 980 | 842 | AT5G55125.2 | Ribosomal protein L31 |
| Phvul.006G184300 | 21836 | 26789 | 9149 | 8706 | AT4G31700.1 | ribosomal protein S6 |
| Phvul.006G208900 | 41193 | 30514 | 9058 | 8075 | AT3G23390.1 | Zinc-binding ribosomal protein family protein |
| Phvul.006G212200 | 3410 | 7548 | 1111 | 1057 | AT1G25260.1 | Ribosomal protein L10 family protein |
| Phvul.007G009400 | 42612 | 23230 | 8133 | 7325 | AT3G09500.1 | Ribosomal L29 family protein |
| Phvul.007G023100 | 362 | 1039 | 156 | 209 | AT1G31817.1 | Ribosomal L18p/L5e family protein |
| Phvul.007G060700 | 36425 | 39077 | 12281 | 11145 | AT4G34670.1 | Ribosomal protein S3Ae |
| Phvul.007G072100 | 51146 | 33803 | 9844 | 8657 | AT5G02450.1 | Ribosomal protein L36e family protein |
| Phvul.007G076200 | 25247 | 28422 | 5771 | 5492 | AT1G33140.1 | Ribosomal protein L6 family |
| Phvul.007G080500 | 31616 | 23499 | 6772 | 5956 | AT1G22780.1 | Ribosomal protein S13/S18 family |
| Phvul.007G081800 | 51036 | 126838 | 29403 | 28551 | AT1G43170.1 | ribosomal protein 1 |
| Phvul.007G083400 | 43114 | 32092 | 9438 | 8301 | AT1G22780.1 | Ribosomal protein S13/S18 family |
| Phvul.007G139500 | 1691 | 1985 | 774 | 626 | AT2G18400.1 | ribosomal protein L6 family protein |
| Phvul.007G146800 | 27649 | 27151 | 8016 | 7244 | AT3G05560.3 | Ribosomal L22e protein family |
| Phvul.007G162900 | 52573 | 68734 | 26669 | 25384 | AT3G09630.1 | Ribosomal protein L4/L1 family |
| Phvul.007G163700 | 32450 | 36934 | 9396 | 8124 | AT5G02960.1 | Ribosomal protein S12/S23 family protein |
| Phvul.007G172400 | 19358 | 16825 | 15928 | 15601 | AT3G63190.1 | ribosome recycling factor, chloroplast precursor |
| Phvul.007G185800 | 24707 | 21927 | 8203 | 7312 | AT5G28060.1 | Ribosomal protein S24e family protein |
| Phvul.007G189900 | 84597 | 50627 | 15313 | 13926 | AT1G04480.1 | Ribosomal protein L14p/L23e family protein |
| Phvul.007G197600 | 13010 | 14179 | 3080 | 2781 | AT3G05560.2 | Ribosomal L22e protein family |
| Phvul.007G223300 | 2453 | 4933 | 1052 | 1063 | AT3G23620.1 | Ribosomal RNA processing Brix domain protein |
| Phvul.007G224900 | 42057 | 23263 | 6524 | 6247 | AT2G36620.1 | ribosomal protein L24 |
| Phvul.007G230200 | 38237 | 29338 | 8868 | 8248 | AT3G44590.1 | 60S acidic ribosomal protein family |
| Phvul.007G230900 | 18862 | 10901 | 3380 | 3179 | AT5G04800.3 | Ribosomal S17 family protein |
| Phvul.007G231100 | 35203 | 22548 | 7328 | 6523 | AT5G04800.3 | Ribosomal S17 family protein |
| Phvul.007G231200 | 42358 | 40370 | 11250 | 9809 | AT5G35530.1 | Ribosomal protein S3 family protein |
| Phvul.007G251500 | 4880 | 5079 | 1893 | 1865 | AT4G35490.1 | mitochondrial ribosomal protein L11 |
| Phvul.007G264200 | 34483 | 33066 | 10635 | 10036 | AT2G37270.2 | ribosomal protein 5B |
| Phvul.007G274200 | 25137 | 32354 | 9226 | 8858 | AT3G62870.1 | Ribosomal protein L7Ae/L30e/S12e/Gadd45 family protein |
| Phvul.007G276800 | 32798 | 33918 | 7726 | 7383 | AT4G27090.1 | Ribosomal protein L14 |
| Phvul.008G015200 | 7192 | 8083 | 1691 | 1548 | AT3G55280.2 | ribosomal protein L23AB |
| Phvul.008G019800 | 41914 | 38868 | 13265 | 11807 | AT1G36240.1 | Ribosomal protein L7Ae/L30e/S12e/Gadd45 family protein |
| Phvul.008G022900 | 646 | 684 | 141 | 138 | AT3G15460.1 | Ribosomal RNA processing Brix domain protein |
| Phvul.008G022950 | 866 | 993 | 172 | 179 | AT3G15460.1 | Ribosomal RNA processing Brix domain protein |
| Phvul.008G041700 | 22323 | 17064 | 4173 | 3756 | AT1G26880.1 | Ribosomal protein L34e superfamily protein |
| Phvul.008G048300 | 1169 | 3118 | 1169 | 1104 | AT3G26360.1 | Ribosomal protein S21 family protein |
| Phvul.008G066200 | 1737 | 2723 | 955 | 867 | AT3G46210.1 | Ribosomal protein S5 domain 2-like superfamily protein |
| Phvul.008G133450 | 48367 | 32316 | 10857 | 10119 | AT5G59240.1 | Ribosomal protein S8e family protein |
| Phvul.008G136100 | 20952 | 36388 | 14278 | 12403 | AT4G18100.1 | Ribosomal protein L32e |
| Phvul.008G150700 | 5828 | 4108 | 1469 | 1355 | AT5G53070.1 | Ribosomal protein L9/RNase H1 |
| Phvul.008G161800 | 1417 | 3350 | 1070 | 903 | AT2G47420.1 | Ribosomal RNA adenine dimethylase family protein |
| Phvul.008G204800 | 25688 | 35492 | 8990 | 8311 | AT5G12110.1 | Glutathione S-transferase, C-terminal-like;Translation elongation factor EF1B/ribosomal protein S6 |
| Phvul.008G221000 | 2141 | 2972 | 1249 | 1028 | AT5G07090.1 | Ribosomal protein S4 (RPS4A) family protein |
| Phvul.008G221300 | 41061 | 57397 | 14089 | 13544 | AT5G07090.1 | Ribosomal protein S4 (RPS4A) family protein |
| Phvul.008G223300 | 15395 | 10565 | 3461 | 3316 | AT2G40510.1 | Ribosomal protein S26e family protein |
| Phvul.008G224500 | 1668 | 3120 | 1240 | 1126 | AT2G40010.1 | Ribosomal protein L10 family protein |
| Phvul.008G227300 | 18112 | 19269 | 6766 | 6139 | AT1G74270.1 | Ribosomal protein L35Ae family protein |
| Phvul.008G255000 | 97214 | 77048 | 22721 | 20463 | AT3G13580.1 | Ribosomal protein L30/L7 family protein |
| Phvul.008G286800 | 3286 | 2857 | 1053 | 785 | AT5G27820.1 | Ribosomal L18p/L5e family protein |
| Phvul.009G024800 | 6028 | 4934 | 1612 | 1612 | AT2G34480.1 | Ribosomal protein L18ae/LX family protein |
| Phvul.009G033000 | 1830 | 1738 | 621 | 552 | AT5G40080.1 | Mitochondrial ribosomal protein L27 |
| Phvul.009G039400 | 30897 | 31281 | 6448 | 6133 | AT1G33140.1 | Ribosomal protein L6 family |
| Phvul.009G039600 | 317 | 408 | 133 | 115 | AT1G33140.1 | Ribosomal protein L6 family |
| Phvul.009G045800 | 51641 | 37385 | 10589 | 9644 | AT5G24510.1 | 60S acidic ribosomal protein family |
| Phvul.009G049400 | 30258 | 26469 | 5498 | 5453 | AT2G36170.1 | Ubiquitin supergroup;Ribosomal protein L40e |
| Phvul.009G089400 | 27584 | 14297 | 4980 | 4350 | AT4G31985.1 | Ribosomal protein L39 family protein |
| Phvul.009G141700 | 19676 | 17377 | 6803 | 6004 | AT2G40510.1 | Ribosomal protein S26e family protein |
| Phvul.009G141800 | 26472 | 17716 | 6758 | 6114 | AT2G40510.1 | Ribosomal protein S26e family protein |
| Phvul.009G167000 | 8822 | 11907 | 3526 | 3183 | AT2G18110.1 | Translation elongation factor EF1B/ribosomal protein S6 family protein |
| Phvul.009G172300 | 45797 | 51668 | 18612 | 16665 | AT5G39850.1 | Ribosomal protein S4 |
| Phvul.009G174700 | 33927 | 27211 | 7501 | 6703 | AT1G70600.1 | Ribosomal protein L18e/L15 superfamily protein |
| Phvul.009G188900 | 3219 | 3029 | 999 | 1007 | AT1G07830.1 | ribosomal protein L29 family protein |
| Phvul.009G214400 | 30109 | 42210 | 9683 | 9095 | AT4G18100.1 | Ribosomal protein L32e |
| Phvul.009G234700 | 12000 | 16829 | 6253 | 5490 | AT3G13580.3 | Ribosomal protein L30/L7 family protein |
| Phvul.009G241700 | 87075 | 47094 | 15608 | 14285 | AT4G15000.1 | Ribosomal L27e protein family |
| Phvul.009G254800 | 16295 | 9587 | 2764 | 2559 | AT3G06680.1 | Ribosomal L29e protein family |
| Phvul.010G024600 | 40788 | 41195 | 15169 | 13975 | AT5G59240.1 | Ribosomal protein S8e family protein |
| Phvul.010G032100 | 14775 | 18651 | 4230 | 4178 | AT5G10360.1 | Ribosomal protein S6e |
| Phvul.010G033200 | 18694 | 19155 | 4624 | 4520 | AT2G05220.2 | Ribosomal S17 family protein |
| Phvul.010G043500 | 61371 | 65719 | 21903 | 20368 | AT1G26910.1 | Ribosomal protein L16p/L10e family protein |
| Phvul.010G044100 | 32743 | 22991 | 6402 | 5842 | AT3G10950.1 | Zinc-binding ribosomal protein family protein |
| Phvul.010G046600 | 2040 | 4563 | 1552 | 1551 | AT2G20060.1 | Ribosomal protein L4/L1 family |
| Phvul.010G068219 | 18553 | 17533 | 6475 | 5381 | AT4G27090.1 | Ribosomal protein L14 |
| Phvul.010G117700 | 49148 | 55739 | 19313 | 18088 | AT5G39740.2 | ribosomal protein L5 B |
| Phvul.010G125100 | 70854 | 80966 | 27131 | 25455 | AT3G04770.2 | 40s ribosomal protein SA B |
| Phvul.010G137600 | 1919 | 2854 | 923 | 970 | AT3G13882.1 | Ribosomal protein L34 |
| Phvul.010G142300 | 62828 | 33784 | 7613 | 6873 | AT4G29410.2 | Ribosomal L28e protein family |
| Phvul.010G160500 | 33877 | 25920 | 9854 | 8562 | AT2G32060.3 | Ribosomal protein L7Ae/L30e/S12e/Gadd45 family protein |
| Phvul.011G031900 | 54299 | 69314 | 16415 | 14895 | AT3G62870.1 | Ribosomal protein L7Ae/L30e/S12e/Gadd45 family protein |
| Phvul.011G043300 | 47535 | 36566 | 12098 | 10291 | AT2G21580.1 | Ribosomal protein S25 family protein |
| Phvul.011G067600 | 26102 | 35267 | 8115 | 7443 | AT3G55280.2 | ribosomal protein L23AB |
| Phvul.011G079400 | 49595 | 64946 | 19011 | 16747 | AT2G37190.1 | Ribosomal protein L11 family protein |
| Phvul.011G126600 | 11449 | 10618 | 3333 | 2992 | AT5G45775.2 | Ribosomal L5P family protein |
| Phvul.011G182132 | 26957 | 24484 | 5980 | 5687 | AT5G45775.2 | Ribosomal L5P family protein |
| Phvul.011G203500 | 2937 | 4663 | 770 | 802 | AT3G01800.1 | Ribosome recycling factor |
| Phvul.L001643 | 14390 | 12262 | 3185 | 2861 | AT2G39390.1 | Ribosomal L29 family protein |
| Phvul.L001941 | 43631 | 34999 | 7632 | 7166 | AT5G02610.1 | Ribosomal L29 family protein |
| Phvul.L002181 | 51062 | 48606 | 17963 | 16491 | AT5G39740.1 | ribosomal protein L5 B |
| Phvul.L002243 | 44630 | 38306 | 9449 | 8724 | AT3G55280.2 | ribosomal protein L23AB |
| Phvul.L003700 | 27503 | 16438 | 6619 | 5429 | AT3G16080.1 | Zinc-binding ribosomal protein family protein |
| Phvul.L003800 | 35519 | 15071 | 5246 | 4541 | AT3G16080.1 | Zinc-binding ribosomal protein family protein |
| Phvul.L003837 | 24803 | 21684 | 6254 | 5703 | AT3G02080.1 | Ribosomal protein S19e family protein |
| Phvul.001G180400 | 1172 | 80 | 1266 | 880 | ATCG00830.1 | ribosomal protein L2 |
| Phvul.003G089801 | 408 | 190 | 1083 | 903 | ATCG00900.1 | Ribosomal protein S7p/S5e family protein |
| Phvul.005G058000 | 10 | 12 | 238 | 108 | AT1G56070.1 | Ribosomal protein S5/Elongation factor G/III/V family protein |

Supplement table 8. A list of primer sequences used in qPCR array.

| Primername | Sequence (5’-3’) |
| --- | --- |
| PIP41-qF | GAGGATGAACTTGCTGATAATGG |
| PIP41-qR | GTCAACTCTAAGCCAGAATCG |
| PvSL1-qF | GCGTCAAAAGCATTTTCCATAAG |
| PvSL1-qR | AGCATTTCCAAGCAATAATTTCAG |
| Phvul.009G021400-qF | GTAGTGGATGCGGAAGTACG |
| Phvul.009G021400-qR  Phvul.009G241100-qF Phvul.009G241100-qR | TGAAGGATGTCAATGTGAGTTGT  AGAATGGAACCTCGGCTACC  GGAAGTTGAACACGCACTC |
